# Programmable gear-based mechanical metamaterials

**DOI:** 10.1038/s41563-022-01269-3

**Published:** 2022-06-09

**Authors:** Xin Fang, Jihong Wen, Li Cheng, Dianlong Yu, Hongjia Zhang, Peter Gumbsch

**Affiliations:** 1 Laboratory of Science and Technology on Integrated Logistics Support, College of Intelligent Science and Technology, National University of Defense Technology, Changsha, China; 2 Institute for Applied Materials, Karlsruhe Institute of Technology (KIT), Karlsruhe, Germany; 3 Department of Mechanical Engineering, Hong Kong Polytechnic University, Hong Kong, China; 4 Fraunhofer Institute for Mechanics of Materials IWM, Freiburg, Germany

**Keywords:** Structural materials, Mechanical engineering

## Abstract

Elastic properties of classical bulk materials can hardly be changed or adjusted in operando, while such tunable elasticity is highly desired for robots and smart machinery. Although possible in reconfigurable metamaterials, continuous tunability in existing designs is plagued by issues such as structural instability, weak robustness, plastic failure and slow response. Here we report a metamaterial design paradigm using gears with encoded stiffness gradients as the constituent elements and organizing gear clusters for versatile functionalities. The design enables continuously tunable elastic properties while preserving stability and robust manoeuvrability, even under a heavy load. Such gear-based metamaterials enable excellent properties such as continuous modulation of Young’s modulus by two orders of magnitude, shape morphing between ultrasoft and solid states, and fast response. This allows for metamaterial customization and brings fully programmable materials and adaptive robots within reach.

## Abstract (web summary)

A design paradigm to create robust robotic metamaterials using versatile gear clusters is demonstrated. It enables intriguing programmability of elastic properties and shape while preserving stability for intelligent machines.

## Main

**Fig. 1:**
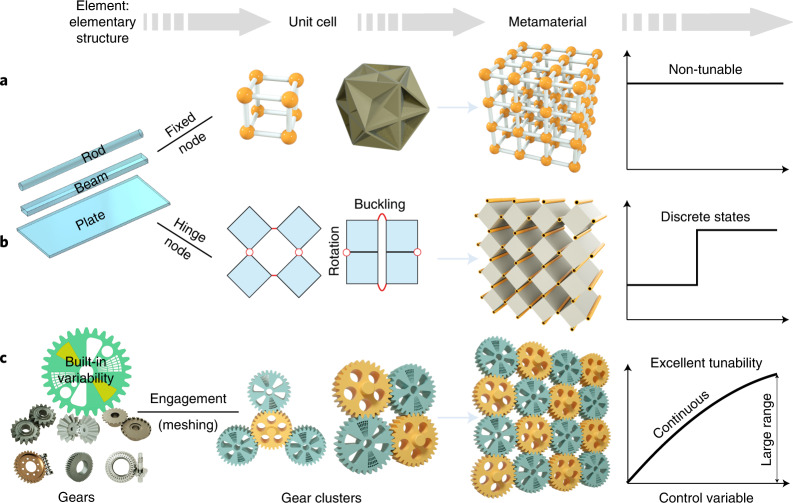
Design concepts of mechanical metamaterials. **a**,**b**, Classical paradigm: non-tunable metamaterial (**a**) and typical reconfigurable metamaterial (**b**). **c**, Gear-based metamaterials.

Materials featuring tunable elastic properties^1,2^ offer tremendous possibilities for smart machines, robots, aircraft and other systems^3–5^. For example, robotic systems with variable stiffness can adapt to missions like grabbing^6^ and jumping, or maintain optimal performance in a changeable environment^7^. However, elastic properties of conventional materials are barely tunable even if phase changes are induced. Mechanical metamaterials^8–11^ are artificial architected materials that exhibit properties beyond those of classical materials^12–16^. Most existing metamaterials integrate monofunctional load-bearing elementary structures (such as rods, beams or plates) in specified topologies with fixed or hinged nodes (Fig. 1). Reconfigurable metamaterials open possibilities for drastic changes in properties^17–19^. When stimulated by stress, heat or electromagnetic fields, reconfigurations in these metamaterials are induced by the formation of new contacts, buckling^20–22^ or rotating hinges^23–25^. Due to node constraints, this permits the reshaping among only a few stable states^26,27^ and often includes unstable states, which limits the tunability. Reducing the connectivity^28^ or relaxing the constraints (for example, with chiral structures^9^ or by connecting elements with flexural traps^29^) can enable more states to improve the shape-changing capability, but this inevitably deteriorates the robustness and structural stability that are essential for most applications. Moreover, reconfiguration, including the shape-memory effect, usually involves large deformation that either leads to irreversible plastic deformation or adversely competes with the commonly required high stiffness^18^. Although the chemical-responsive materials^30,31^ enable some in situ tunability, the regulation process of their elastic properties is usually very slow, just like for thermal-responsive materials^19^. Assembling rods mounted with gears into special lattices can improve the stability while preserving the rotatable nodes^32^, but engineering practical and robust metamaterials with continuously tunable elasticity, especially with fast in situ tunability in service, remains a major challenge.

## Design concept

Overcoming these challenges requires an unprecedented design paradigm. First, tunability may be realized by assembling elements with built-in stiffness gradients. Second, the coupling between elements must comply with large deformation. Achieving tunable yet strong solids requires ensuring tunability under large force and robust controllability while avoiding plastic deformation in tuning. We find that such a mutable-yet-strong coupling can be realized with gear clusters. Gears provide an ideal mechanism to smoothly transmit rotation and heavy compressive loads thanks to the reliable gear engagement (meshing). Stiffness gradients can be built into an individual gear body or realized with hierarchical gear assemblies. Gear clusters can be assembled into manifolds and can, as metacells, be periodically arranged to form metamaterials (Fig. 1c). The proposed design concept is very general since there exist numerous architectures for gear assembly. Exotic functionality and flexible tunability can emerge from the diversity of gear types, built-in variability and cluster organization. We create several metamaterial prototypes with different gear clusters to demonstrate this.

## Metamaterial based on Taiji gears

**Fig. 2:**
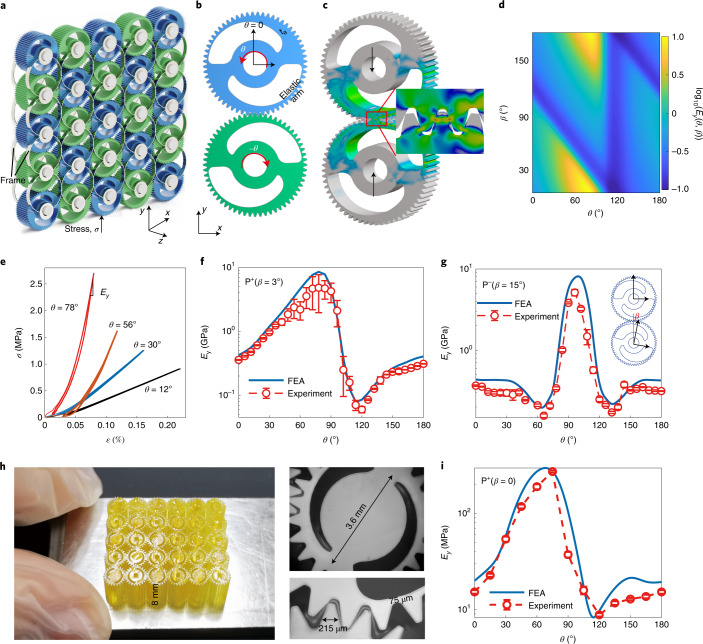
Mechanical metamaterial based on Taiji gears. **a**, Metamaterial architecture. The blue and green colours show different orientations of gears. **b**, A pair of meshing gears for P^+^(3°). The *x*, *y* and *z* denote the global coordinates; the origin of the local cylindrical coordinates for spin rotation *θ* is set at the gear centre. *t*_a_, the thickness of the arm. **c**, Typical Mises stress contour of a meshing pair for *θ* = 0 and compressive strain *ε* = 0.18%. Deformation is enlarged 20 times. **d**, Numerical value of log_10_(*E*_*y*_(*θ*, *β*)) for P^+^(*β*). **e**, Measured loading–unloading stress–strain curves for metamaterials with different rotation angles *θ*. **f**,**g**, Young’s modulus as a function of the gear rotation angle *θ* for positive P^+^(3°) and negative P^−^(15°) polarities, respectively. Inset: the meshing pair for P^−^(15°) **h**, Directly printed micro metamaterial consisting of P^+^(0°) 5 × 6 Taiji gears, with close-up images showing dimensions. **i**, *E*_*y*_(*θ*) of the P^+^(0°) micro specimen. The error bars and the average values in **f**,**g** and **i** are evaluated by choosing different intervals along the curve in **e**.

The first prototype is created using compactly coupled periodic gears and two lattice frames (front and back) to arrange the gears into a simple quadratic pattern (Fig. 2a). The plane gears contain hollow sections. The outer part forms two elastic arms whose radial thickness smoothly varies with the rotation angle *θ* (Fig. 2b and Supplementary Figs. 1 and 2). Subject to compressive loading, deformation in the arms is dominated by bending (Fig. 2c). The effective stiffnesses of both an arm *k*_arm_ and a pair of arms *K*_p_ depend on the angle *θ* (Supplementary Fig. 3). The homogenized Young’s modulus of the metamaterial along the *y* axis *E*_*y*_ = *K*_p_/*B* + *E*_f_ has contributions from the gears and frame (Methods, ‘Equivalent method’ section). Here, *E*_f_ is the stiffness of the frame and *B* denotes the gear width. *E*_*y*_ is continuously tunable by rotating the gears and dominantly depends on *K*_p_ since min(*K*_p_/*B*) ≫ *E*_f_ = 2.06 MPa here.

The tunability relies on the shape of the built-in hollow section. Among diverse choices, the shape inspired by the Chinese Taiji diagram (Fig. 2b), characterized by a spiral direction, can give smooth variation and polarity. The angle difference between the two local coordinates is *β* (Fig. 2g). The spin rotations are opposite in any two meshing gears. Also, the spiral directions of the Taiji patterns on the front and the back faces are reversed. Therefore, the meshing mode of a pair of gears has two polarities. When the spiral directions of patterns are opposite (Fig. 2b), the polarity is positive, labelled as P^+^(*β*). The meshing pair in Fig. 2g features negative polarity, P^−^(*β*).

We employ finite element analysis (FEA) to simulate the contact problem in gear-based metamaterials (Methods and Supplementary Figs. 4 and 5). Contact nonlinearity becomes apparent for high *K*_p_ (Supplementary Fig. 3). Young’s modulus is evaluated from the slope of the uniaxial stress–strain curves at relatively large strain *ε*. An all-metallic prototype, consisting of 5 × 5 copper gears and steel frames, is manufactured and assembled with P^+^(3°) and P^−^(15°) metacells, respectively (Supplementary Fig. 2). The gear has 60 teeth, with the tooth thickness *t*_to_ = 0.35π mm, diameter *D* = 42 mm and width *B* = 20 mm. Measured cyclic loading–unloading curves show some hysteresis (Fig. 2e). This is ascribed to the sliding friction between the meshed teeth (Supplementary Fig. 6). Figure 2f,g demonstrates that experimental results of *E*_*y*_(*θ*) are in excellent agreement with FEA. The modulation period is 180° in both the P^+^(3°) and P^−^(15°) cases. The smooth *E*_*y*_(*θ*) curve indicates that the obtainable stable states are dense and that continuous tunability is achieved. Both polarity and *β* affect the tunable range and the correlation between the tunable properties and *θ* (Fig. 2d). For P^+^(3°) in Fig. 2f, the zigzag curve of *E*_*y*_(*θ*) reaches the maximum value *E*_max_ = 7.67 GPa at *θ* = 78°, where the solid parts of the gears are in contact, and a minimal value *E*_min_ = 0.102 GPa at *θ* = 114°, where the meshing connects the forearms. This experimentally obtained modulation range of *E*_max_/*E*_min_ = 75 demonstrates the spectacular reconfigurability. For P^−^(15°), *E*_y_(*θ*) is sombrero-shaped with a tunable range of 33 from *E*_min_ = 0.156 GPa to *E*_max_ = 5.13 GPa. Since *E*_*x*_(*θ*) = *E*_*y*_(*θ* + 90°), the anisotropy in orthogonal directions also changes with *θ*. For example, the P^+^(3°) metamaterial can be continuously modulated from *E*_*x*_ = *E*_*y*_ to the maximum ratio (*E*_*y*_/*E*_*x*_)_max_ = 24.8. The latter gives a metamaterial with negligible lateral expansion upon compression (Supplementary Fig. 7). Compared to existing designs, the node constraints in gear-based metamaterials are relaxed, but the connection stability and reconfiguration robustness are maintained at any *θ* even under large compressive loads (Supplementary Video 1). The design is also robust to accommodate manufacturing inaccuracies when regarding the angle *β* as an indicator of the alignment error of the gears. Figure 2d shows that the programmability is preserved even for large *β*.

The all-metallic metamaterial introduced above is manufactured by assembling individual gears. For scale-up and miniaturization, it is desirable to avoid the assembly of individual parts. Next, we demonstrate that integrated gear-based metamaterials can be directly manufactured with three-dimensional (3D) printing, even on the microscale. The major challenge for such integrated manufacturing is to guarantee that the meshing teeth are not fused together but still reliably engaged. To tackle this problem, a small clearance is reserved between the surfaces of the meshing teeth in the assembled digital model to overcome manufacturing errors (Methods). Here we manufacture an integrated micro metamaterial consisting of 5 × 6 Taiji gears (Fig. 2h) by adopting the projection micro-stereolithography 3D printing technique. The diameter and tooth thickness of the Taiji gear are 3.6 mm and 235 µm, respectively; the thickest arm is 75 µm (Supplementary Fig. 8). The micro gears are arranged with P^+^(0°), and the reserved minimal clearance between the teeth is 32 µm. The sample is made of a photosensitive resin with a Young’s modulus of 3.5 GPa. As experimentally demonstrated in Fig. 2i, the equivalent modulus *E*_*y*_(*θ*) of this micro specimen can be smoothly tuned by 35 times (from 8.3 MPa to 295 MPa). Using this integrated design strategy, gear-based metamaterials could be scaled up in size and number of gears with appropriate high-resolution large-scale 3D printing facilities. Modulation of such integrated metamaterials can be achieved with distributed drives or motors (Supplementary Video 2 and Supplementary Fig. 9a).

## Metamaterial based on planetary gears

**Fig. 3:**
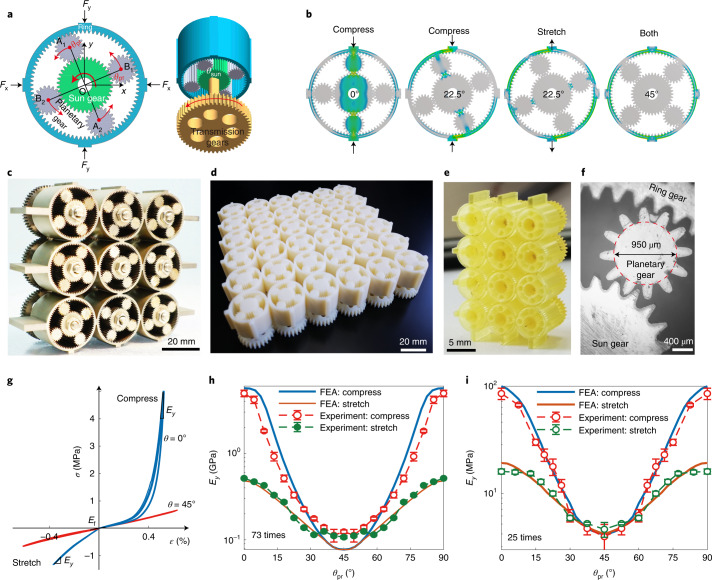
Metamaterial consisting of planetary gear systems. **a**, Metacell, a planetary gear system. The origin (O) of the coordinates *x*,*y* is the centre of the sun gear (green). A_*i*_ and B_*i*_ denote the centres of planetary gears (grey). Their revolution angle denotes *θ*_pr_ = ∠YOA_1_. The case A_1_A_2_ ⊥ B_1_B_2_ is drawn here, and thus *θ*_pr_ = ∠XOB_1_ = ∠YOA_1_. A transmission gear (yellow) connects to the sun gear through a shaft. **b**, Typical compressive and tensile deformation of a metacell. **c**, Macro metallic metamaterial architecture. **d**, Macro polymer metamaterial with 6 × 6 metacells. **e**, Micro polymer metamaterial with 3 × 4 metacells. **f**, Micrograph for the metamaterial in **e**. Specimens in **d** and **e** are fabricated with integrated manufacturing. **g**, Measured strain–stress curves. **h**,**i**, Measured and simulated Young’s moduli under compression and tensile deformation, *E*_c_ and *E*_t_, of the macro metallic (**h**) and micro (**i**) specimens. Results of the macro polymer sample are shown in Supplementary Fig. 12b. The error bars and the average values in **h** and **i** are evaluated by choosing different intervals near the maximum strain along the curve in **g**.

Obviously, this first metamaterial is tunable only under compressive loading. The tensile load is carried by the frame, and the tensile modulus is *E*_t_ = *E*_f_. One may also aim at strong metamaterials whose compressive and tensile moduli are both tunable while preserving structural integrity. This can be achieved by organizing a planetary gear system as a metacell (Fig. 3a). In this example, the metacell contains six gears: an inner-toothed ring gear (Supplementary Fig. 10), a central sun gear and two pairs of planetary gears A_1_–A_2_ and B_1_–B_2_. Gear centres A_1_–O–A_2_ (and B_1_–O–B_2_) are colinear. Using this gear cluster, we create a hierarchical and strong metamaterial whose tunability emerges from the relative rotation of the gears inside the metacell. The thickness of the ring *t*_r_ is uniform. Neighbouring rings are rigidly connected in a quadratic lattice, which ensures structural integrity. Planetary gears revolve along the ring when rotating the sun gear by *θ*_sun_. Their position is given by the revolution angle *θ*_pr_ = *θ*_sun_*r*_sun_/(*R*_in_ + *r*_sun_), where *R*_in_ and *r*_sun_ are the radii of the ring and sun gears, respectively (Supplementary Table 1 for parameter values). The teeth prevent relative slippage between the two gears even under tension. The metamaterial elastic properties are given by the effective stiffness of the annulus ring supported by the planetary gears that act as fulcrums (Fig. 3b). The position *θ*_pr_ of the planetary gears determines the elastic properties. We adopt the orthogonal relation A_1_A_2_ ⊥ B_1_B_2_, which gives a large tunable range and symmetric behaviour with a modulation period of 90°. The tunable range can be further modified using the angle ∠A_1_OB_1_. For the assembled metamaterial, all sun gears are connected to transmission gears by shafts (Fig. 3a), and those transmission gears are compactly coupled. Thereby robust reconfiguration of all metacell patterns can be achieved by rotating transmission gears.

*F*_*x*_ and *F*_*y*_ denote the compressive loads in the *x* and *y* directions, respectively (Fig. 3a). Under uniaxial compression (*F*_*y*_ > 0, *F*_*x*_ = 0), only the pair of planetary gears with an angle smaller than that of the loading axial (min(∠YOA_1_, ∠YOB_1_) < 45°) supports the load (Fig. 3b). Stress in the other pair is zero. Conversely, under uniaxial tension, only the other pair is load-bearing. The two pairs exchange roles at *θ*_pr_ = 45°, and the material is orthogonally isotropic. This metamaterial presents a more remarkable compressive nonlinearity because four pairs of meshing teeth in a metacell bear loads. Both the compressive and tensile moduli *E*_c_ and *E*_t_ reach maxima at *θ*_pr_ = 0, but $E_{{{\mathrm{c}}}}^{{{{\mathrm{max}}}}} \gg E_{{{\mathrm{t}}}}^{{{{\mathrm{max}}}}}$, and thus the static compressive-tension symmetry is broken (Fig. 3g). Moreover, at *θ*_pr_ = 45°, no stress is transmitted to the planetary gears (Fig. 3b); both moduli reach minima there, and $E_{{{\mathrm{c}}}}^{{{{\mathrm{min}}}}} = E_{{{\mathrm{t}}}}^{{{{\mathrm{min}}}}}$.

We fabricate three kinds of specimens using this strategy. An all-steel macro metamaterial is manufactured by assembling 3 × 3 metacells (Fig. 3c) with lattice constants *a*_*x*_ = *a*_*y*_ = 27 mm. The steel gears have a small tooth thickness *t*_to_ = 0.15π mm, and *R*_in_ = 12 mm, *r*_sun_ = 6 mm and *t*_r_ = 1 mm. Integrated manufacturing of this prototype is more challenging than that of the Taiji pattern because there are two layers and every metacell possesses eight pairs of meshing teeth. The integrated prototype can also be directly manufactured by 3D printing, at both macro and micro scales (Methods, Supplementary Figs. 11 and 12 and Supplementary Video 3 for more details). We print a 6 × 6 macro specimen (Fig. 3d) using a polymer with a Young’s modulus of 2.5 GPa, and print a 3 × 4 micro specimen (Fig. 3e) using a resin with a Young’s modulus of 3.5 GPa. The size and the number of metacells are limited by the capability of the 3D printer rather than the design strategy. The micro polymer sample (Fig. 3e) has *R*_in_ = 2.4 mm, *r*_sun_ = 0.6 mm, *t*_r_ = 0.3 mm and *a*_*x*_ = *a*_*y*_ = 5.4 mm, with a tooth width and height of 135 µm and 225 µm, respectively.

The experimental results are consistent with the FEA simulations for all specimens (Fig. 3g–i and Supplementary Fig. 11). In this strong hierarchical metamaterial, we can smoothly tune the compressive modulus *E*_c_ of the macro metallic specimen by 46 times (5.2–0.11 GPa), the macro polymer specimen by 55 times (69–1.25 MPa; Supplementary Fig. 12b) and the micro specimen by 25 times (100–4 MPa). Meanwhile, their tensile modulus *E*_t_ can be tuned by 5 times (0.52–0.11 GPa), 5.6 times (7–1.25 MPa) and 5 times (20–4 MPa), respectively. In Fig. 3h, some differences between experiment and FEA near *θ*_pr_ = 45° arise from the boundary conditions (Supplementary Fig. 13). The in situ tunability combined with the reasonably large moduli in tension and compression as well as large shear rigidity makes this metamaterial design particularly robust and strong, yet tunable. Furthermore, the metamaterials can be synchronously controlled with distributed motors at both the macro and micro scales (Supplementary Video 4).

## Mechanisms for stability

**Fig. 4:**
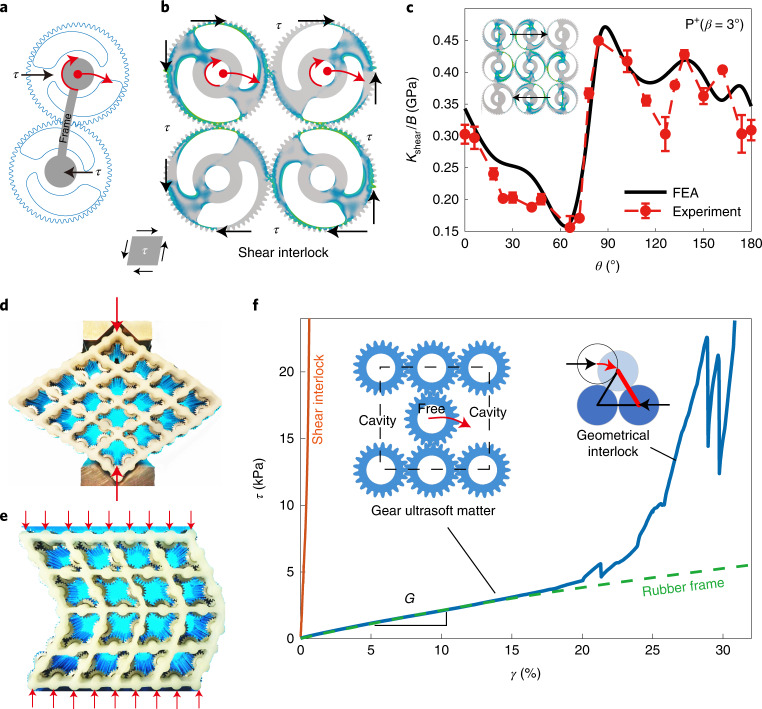
Strong or ultrasoft metamaterials under shear. **a**, Schematic shear deformation of a meshing pair. **b**, Four meshing gears in the state of shear interlock, *θ* = 60°. The circular arrows show the planetary and spin rotations, which block each other. Periodic boundary conditions are applied on this metacell (Supplementary Fig. 14). **c**, Theoretical and experimental shear stiffness *K*_shear_/*B* under shear interlock. The 3 × 3 architecture with P^+^(3°) is studied here (Supplementary Fig. 15). The stiffness is strong (>150 MPa) and narrowly tunable (~3 times). The error bars and the average values are evaluated by choosing different intervals along the tested strain–stress curves. **d**,**e**, Real-life photographs for the deformation modes of the prototype under diagonal and surface compressions, respectively. The light blue, white and dark blue parts are the background, rubber frame and gears (**d**,**e**). **f**, Experimental strain–stress curves for the shear interlocked metamaterial, the ultrasoft gear matter and the rubber frame by itself. Inset: three blue disks illustrate the geometrical interlock, which occurs when three meshing gears form a closed triangle.

Interestingly, the metamaterial in Fig. 2a (a discrete gear lattice with a very soft frame) remains stable under compressive stresses and shows large rigidity in shear. One of the contributing factors underpinning the observed stability stems from the non-uniform loading of the meshing teeth at different points, which leads to bending deformations that tightly grip the teeth together (Supplementary Fig. 5). The relatively large shear modulus of the metamaterial, *G* = *G*_g_ + *G*_f_, is composed of the shear moduli generated by gears (*G*_g_) and by frames (*G*_f_ = 1.04 MPa). Shear force induces both spin and the planetary rotation of gears. For a pair of gears, the relative planetary rotation leads to zero shear resistance, *G*_g_ = 0, giving a highly unstable state (Fig. 4a). However, in a group of four gears (shown in Fig. 4b), shear stress *τ* induces mutual locking of the planetary rotation by the opposite spin of the neighbouring gears, which is referred to as shear interlock. We calculate the shear stiffness of the metacell with periodic boundary conditions and the finite *n* × *n* gear lattice (Supplementary Figs. 14–16). Owing to the shear interlock, the shear modulus is large but only marginally tunable (Supplementary Fig. 16), which is demonstrated by the measured generalized shear stiffness *K*_shear_/*B* of the finite 3 × 3 architecture in Fig. 4c.

## Gear metamaterial for shape morphing

The programmability of gear-based metamaterials is not limited to elastic constants. Removing every second gear in every second row of the metamaterial in Fig. 2 can release the shear interlock (inset in Fig. 4f) to generate a state with *G*_g_ = 0. The effective shear modulus is then determined solely by the low stiffness frame, and the metamaterial can be considered as ultrasoft matter. The vanishing shear modulus enables complex deformation modes (Fig. 4d,e and Supplementary Video 5), conducive to shape morphing. To verify this, an ultrasoft prototype consisting of 4 × 4 metacells with rubber frames is manufactured and tested (Fig. 4f). The gears are made of aluminium alloy (Supplementary Fig. 17). Shear tests on the prototype give a tiny modulus of *G* = 21.52 kPa. Independent measurement of the frames gives *G*_f_ = 21.11 kPa, so that *G*_g_ = *G* *–* *G*_f_ is indeed negligibly small. Moreover, the modulus *G* remains tiny until the shear strain *γ* reaches 25%, at which point the semi-free gear interacts with two other gears, which builds a new meshing connection among the three gears. The new connection supports high shear stresses and leads to a sharp rise in *G*, switching the soft matter to a stiff solid. The resulting solid represents a geometrically interlocked state (Methods). Oscillations of the shear stress in Fig. 4c arise from the critical meshing state among the three gears before they interlock (Supplementary Fig. 18). The shear strain at which the geometrical interlock occurs can be adjusted by the size of the neighbouring gears, which in turn determines the limiting (strong) states of a shape-morphing structure. Previous metamaterial designs with vanishing shear modulus, like pentamode metamaterials^33^, show vanishing moduli only at small strains and in extremely fragile structures.

## Potential applications

Conventional machines generally rely on materials with constant stiffness and therefore show constant stiffness themselves. The designed stiffness is then a compromise among stability, safety, efficiency and performance, thus hindering the pursuit of the best performance and efficiency in variable environments. Programmable materials featuring tunable elastic properties, including active mechanical metamaterials, are much anticipated in intelligent machines and systems^1,2^. Here we offer a comparison of typical material designs from the literature^2,19^.

**Fig. 5:**
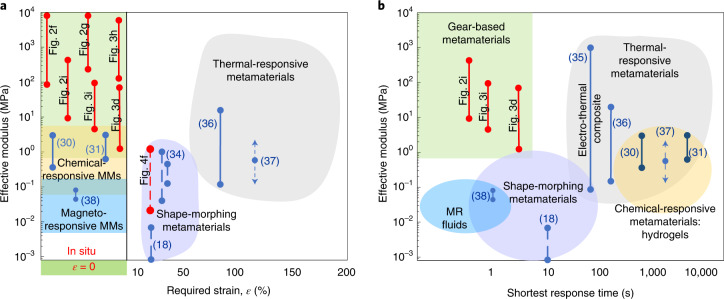
Properties of active mechanical metamaterials. **a**,**b**, Tunable effective modulus versus the required strain *ε* (**a**) and shortest response time (**b**) for tunability. The shaded colour regions (rectangles and ovals) represent the possible tunable ranges of different tunable materials or metamaterials. Pale green, gear-based metamaterials; yellow, chemical-responsive metamaterials; blue, magneto-responsive metamaterials, brown: shape-morphing metamaterials; grey, thermal-responsive metamaterials. These regions partially overlap. The straight lines indicate the tunable ranges. The solid (dashed) lines represent the continuous (non-continuous) tunability. Arrows represent uncertainties. Numbers in parentheses denote the references. The green shaded region at the bottom in **a** signifies that the required strain is zero, that is, the in situ tunability. MMs, metamaterials; MR, magnetorheological. Properties of our gear-based metamaterials are shown by red lines and are also labelled by figure numbers.

The response time, stability, force and energy required for property changes are all critical attributes for variable-stiffness structures^4^. We take the strain and response time required to accomplish a tunable period of material stiffness as metrics to position our gear-based metamaterials among the existing active materials (Fig. 5). Shape-morphing metamaterials^18,34^ enable tunability between or among two or a few stable states. The achieved tunability is non-continuous and requires a large deformation (*ε* ≈ 30%). Thermal-responsive composites^35^ made of shape-memory alloys or polymers may give a continuously tunable modulus. However, they require a long response time, and some suffer from nearly 100% strain^36,37^. Chemical-responsive materials^30,31^ containing hydrogels can offer continuous and in situ tunability, but also require hours of response time. Conventional magneto- or electro-responsive metamaterials^38^ based on elastomers or magnetorheological fluids can give fast, continuous, but narrow tunability, which usually requires a high active voltage (~5 kV) and complex facilities^19^. Our gear-based metamaterials in Figs. 2 and 3 can offer a fast response and the desired broad-range, continuous and in situ tunability of stiffness.

We propose several scenarios to showcase the broad application potential of the proposed gear-based metamaterials in Supplementary Figs. 19–21 and Supplementary Table 2. For robots, a tunable-stiffness leg/actuator can offer high stiffness to stably support a heavy load while walking and a low stiffness for shock protection while jumping or running^4,39^ (Supplementary Figs. 19 and 20). A similar tunable-stiffness isolator is desired in the aero-engine pylon system to maintain the best performance and efficiency at different flight stages (Supplementary Fig. 21). Moreover, the fast-response gear-based metamaterial may give rise to a sensitive variable-stiffness skin, which has been attracting wide attention^40^. Furthermore, resonators with tunable stiffness are critical components in programmable metamaterials for wave manipulation^41,42^. Therefore, gear-based programmable metamaterials can aid in the realization of extensive intelligent machines. In contrast with conventional methods, the programmability enabled by a gear-based metamaterial does not require large deformation and heavy controlling systems, such as hydraulic/pneumatic or magnetic systems, and thus benefits the miniaturization and integration of machines and can even be used in harsh environments such as outer space.

## Conclusions

We show that gear-based mechanical metamaterials provide in situ tunability while preserving stability, strength and high load-bearing capacity. The programmability is robust and easily implementable. Gear clusters provide a vast design space that permits customizable performance of the metamaterials. Besides the demonstarted Young’s modulus, shape morphing and shock protection, the tunability can be extended to other elastic properties like shear modulus, Poisson’s ratio, strength, deformation modes and even damping coefficient (Supplementary Fig. 21). One can also envision 3D metamaterials by using bevel gears, assembling planar gears into hierarchical configurations as in Fig. 3 or synthesizing different types of gear (Supplementary Fig. 22). Integrated manufacturing bridges these tunable properties to produce robust multipurpose devices^43^. With the example of micro metamaterials, further miniaturization and an extension of gear-based metamaterials are possible with high-resolution and large-scale 3D printing.

In conclusion, this work proposes and demonstrates an unconventional design paradigm for programmable dynamic metamaterials via the mutable-yet-strong coupling and built-in variability of gears. We establish the general concept, conceive prototypes, conduct mechanical analyses, demonstrate the flexible tunability and integrated manufacturing at both the macro and micro scales and showcase the broad potential applications. The proposed design paradigm broadens the horizon for designing fully programmable materials, thus offering an impetus to their exploration for practical applications.

## Methods

### Integrated manufacturing

The printer used for the projection micro-stereolithography micro metamaterial fabrication is a BMF NanoArch S130, with a precision of about 5 µm. The material used in microscale 3D printing is a photosensitive resin with a Young’s modulus of about 3.5 GPa. The manufacturing process for the integrated micro metamaterial sample consisting of 5 × 6 Taiji gears follows three steps. First, the assembled gears are printed on a baseplate; those gears are adhered to the plate. Second, the sample is wrapped in a box to constrain the motion of the gears (Supplementary Fig. 9a). Last, everything including the box is removed from the plate. The box with a frame helps maintain the relative angle of the assembly in the removal process.

For the metamaterial based on planetary gears, the layer of the planetary gears is printed first (Supplementary Fig. 12a and Supplementary Video 3). Except for the preserved clearance, the connection shaft between the transmission gear and the sun gear is conical at both the macro and micro scales (Supplementary Fig. 10), which ensures that every printing part, especially the teeth of the transmission gears, is tightly attached on the formed structure. Otherwise, the teeth could move and then fuse together during the printing. At the macro scale, the integrated model is printed with two photosensitive resins using polymer injection with the printer Stratasys Objet260, with a precision of about 50 µm. The stiff model material is wrapped in the soft, soluble support material. The metamaterial acquires the targeted tunability after removing the support material. At the microscale, the material is immersed in the fluid resin during the printing. No support material/structure is required for this model owing to the conical shaft and high precision of projection micro-stereolithography. The sample shown in Fig. 3d is printed with a resin (polymer) with a Young’s modulus of 2.5 GPa.

In integrated manufacturing, the clearance reserved between the surfaces of meshing teeth in the assembled digital model depends on the precision of the printer, the structure and the materials. The minimal clearance *Δ* should be higher than the printer’s precision *p* (manufacturing errors) but much smaller than the tooth height (*h*_t_ = 2.25*m* for standard gears), where the gear module *m* denotes the ratio between the gear diameter *D* and the number of teeth *z*, *m* = *D*/*z* (see Supplementary Text). Here, the minimal clearance between meshing teeth in all macro specimens printed with Objet260 is 86 µm. The minimal clearance for the micro metamaterial consisting of Taiji gears is set to 32 µm, and that for the micro planetary gear-based metamaterial is 21 µm. These clearances are sufficient to alleviate the manufacturing uncertainties to keep the meshing teeth separated but reliably engaged. Based on our 3D printers and tests, we suggest *Δ* > 1.5*p* and *Δ* ≤ *h*_t_/10 = 0.225*m*. This requires *p* < 0.15*m*, which helps us determine the required precision scale with a specified gear size.

### Actuation

As shown in Supplementary Video 2, we prepare a microscale sample consisting of 5 × 5 Taiji gears to show its actuation process. They are embedded into a box, and those gears connect to the frames through micro shafts. The sample is synchronously driven by four d.c. brushless motors (8 mm diameter) connected to the 1 × 1st, 1 × 4th, 4 × 1st and 4 × 4th gears. Here *n* × *m* denotes the position at the *n*th row and *m*th column in the array. As shown in Supplementary Video 4, the macro metamaterial in Fig. 3d is synchronously actuated by four-step motors whose diameter is 20 mm. These motors are synchronously controlled by an electronic controller. The revolving speed of the step motor depends on the impulse frequency generated by the controller. Similarly, the micro sample in Fig. 3e is put in a box and actuated by five micro step motors whose diameter is 5 mm. The controller is identical to the one used for the macro sample.

### FEA

FEA simulations are carried out with the commercial software ANSYS. We compare the accuracy of different finite element models, including two-dimensional (2D), 3D, linear and nonlinear models. The plane stress state is considered in the 2D model. In the linear models, the meshing points of gears are bonded by fixing together the two surfaces in contact, resulting in a linear stress–strain relationship. In the nonlinear models, the size of the contact area on the tooth surface at the meshing points depends on the load, and there is a relative sliding between the contact surfaces. The sliding induces frictional damping if the coefficient of friction is non-zero. We also use a simplified model by removing all teeth, where the contact between two gears becomes that between two cylinders. In principle, the 3D nonlinear model should be the most realistic representation of the experimental set-up. Supplementary Fig. 4 demonstrates that the 2D nonlinear model is in excellent agreement with the 3D nonlinear model. The two linear models produce a large discrepancy with the nonlinear ones, although they still can capture the general variation trend. The simplified model approximately presents the standard results. To enhance the simulation efficiency, we use the 2D nonlinear models in most cases. The 3D model is adopted only when considering the frictional contact.

Our metamaterials embrace a periodic architecture. To evaluate the homogenized elastic and shear moduli, ideal periodic boundary conditions are applied on the unit cell in the FEA. Boundary conditions depend on the deformation mode of the unit cell. The homogenized strain vector is *ε* = (*ε*_*x*_, *ε*_*y*_, *γ*). These strains are realized by enforcing the displacement fields (*u*, *v*) in the plane stress state.

As explained in Supplementary Fig. 14, two types of boundary condition are considered when calculating the shear modulus in the shear interlock state. To show the shear state of a finite *n* × *n* gear lattice, we fix the lower row/column of the gears and apply a displacement field to the upper row/column of the gears. As a second method, periodic boundary conditions are applied on a metacell to calculate the shear modulus. These periodic boundary conditions present the shearing state *ε* = (0, 0, *γ*). In both cases, the strain energy density *W* = *Gγ*^2^/2 is extracted to evaluate the shear modulus *G*. For the *n* × *n* finite structure without periodic boundary conditions, although the equation of the generalized shear stiffness *G*′ = *K*_shear_/*B* is the same as the formula for shear modulus *G* = *τ*/*γ*, the value of *G*′ may not equal the real shear modulus *G* (Supplementary Fig. 15) due to the free edge effects in finite structures (Supplementary Figs. 13a and 14a).

For the metamaterial based on a planetary gear system, the load is applied on the four blocks of the ring. For a metacell in FEA, we specify the uniaxial deformation *v* = *ε*_*y*_*a*_*y*_ and make *ε*_*x*_ free for solving *E*_*y*_.

### Equivalent method

For the metamaterial based on Taiji gears, the deformation mode for meshing gears can be represented by the overall stiffness of a pair of meshing elastic arms *K*_p_ = 1/(1/*k*_arm1_ + 1/*k*_arm2_ + 1/*k*_tooth_) (Supplementary Fig. 3 for their definitions). The stiffnesses of the two arms *k*_arm1_ and *k*_arm2_ are independent of the compressive deformation. As shown in Supplementary Fig. 5, the meshing of a pair of teeth features a line of contact on their surfaces. With compression, a small contact area is generated near the line where sliding occurs during the process. Therefore, the contact stiffness of the teeth *k*_tooth_ depends on the contact pressure on the involute teeth. A high pressure leads to significant contact nonlinearity and results in a dependence of *K*_p_ on the displacement/load. By contrast, deformation mainly occurs in the elastic arm rather than the teeth if *k*_arm_ ≪ *k*_tooth_, and *K*_p_ is constant in this case. The homogenized Young’s modulus in the *y* direction of the metamaterial is *E*_*y*_ = *K*_p_/*B* + *E*_f_. The equivalent methods for shear modulus are explained with Supplementary Fig. 14.

For the metamaterial consisting of a periodic planetary gear cluster, the Young’s modulus depends on the deformation of the ring. The influences of contact nonlinearity between teeth on *E*_*y*_ are the same as described above.

### Geometrical interlock

In a meshing pair, the rotation directions of the driving and driven gears are opposite. In a group of gears, if every meshing is viewed as a connection line, *n* gears form a closed polygon as shown in Fig. 4f. If *n* is odd, spin rotation is incompatible, leading to the locking among the gears. This meshing state is referred to as geometrical interlock.

### Mechanical tests for Young’s modulus

When measuring the Young’s modulus *E*_*y*_ in the metamaterial based on Taiji gears, a compressive load *F*_*y*_ is applied and released from the top of the prototype in Fig. 2a. We control the strain *ε* for different *θ* to overcome clearance nonlinearity while avoiding plastic deformation. The rotation angle *θ* is manually controlled. Similar cyclic loading–unloading tests are performed for the measurement of the shear modulus.

The experimental setting for the test on the metamaterial based on planetary gear systems is shown in Supplementary Fig. 9. When measuring the compressive modulus, a compressive load is applied on the top and the bottom blocks on the rings; when measuring the tensile modulus, we fix the tails on the sample to a pair of clamps and apply tensile loads through the tails.

As shown in Figs. 2e and 3g, the cyclic loading–unloading process features high repeatability, thus testifying to the experimental accuracy. Moduli *E*_*y*_ and *G* are both calculated as the slope around the maximum *ε*. The initial cycle is excluded when fitting *E*_*y*_ and *G*. The choice of the strain interval for the slope calculation affects the final modulus value. The error bars and the average values are evaluated by choosing different intervals along the curve.

### Mechanical tests for shear stiffness

For the metamaterial based on Taiji gears, a sample consisting of 3 × 3 gears and steel frames is manufactured for the measurement of the shear stiffness in the shear interlock state, as shown in Supplementary Fig. 15. A fixture apparatus is fabricated to obtain the shearing state. For the shape-morphing metamaterial, the sample is put in two right-angle grooves, and the load from the testing machine directly transfers to the sample.

## Online content

Any methods, additional references, Nature Research reporting summaries, source data, extended data, supplementary information, acknowledgements, peer review information; details of author contributions and competing interests; and statements of data and code availability are available at 10.1038/s41563-022-01269-3.

## Supplementary information

Supplementary Information Supplementary Figs. 1–23 with explanatory text, Tables 1 and 2 and legends for Videos 1–5.

Supplementary Video 1 Introduction of the metamaterial based on Taiji gears. This video shows the robust tunability and high stability of the proposed metamaterial under a large force.

Supplementary Video 2 Actuation of the micro metamaterial consisting of Taiji gears.

Supplementary Video 3 Integrated 3D printing process of the macro metamaterial consisting of a planetary gear system.

Supplementary Video 4 Actuation of the macro metamaterial consisting of planetary gears.

Supplementary Video 5 Shape-morphing metamaterial. This video shows the structure, manufacturing process and protected shape morphing of this metamaterial.

## Data Availability

The main data and models supporting the findings of this study are available within the paper and Supplementary Information. Further information is available from the corresponding authors upon reasonable request.
