# Supplementary material for: Programmable gear-based mechanical metamaterials
Source: Nat Mater. 2022 Jun 9;21(8):869–76. doi: 10.1038/s41563-022-01269-3 (PMC9345786; doi:10.1038/s41563-022-01269-3)
Supplement: Supplementary file 1 — Supplementary Figs. 1–23 with explanatory text, Tables 1 and 2 and legends for Videos 1–5. [file 41563_2022_1269_MOESM1_ESM.pdf]

nature portfolio

https://doi.org/10.1038/s41563-022-01269-3

Supplementary information

Programmable gear-based mechanical metamaterials

In the format provided by the authors and unedited

Supplementary Information

Programmable gear-based mechanical metamaterials

Xin Fang[1,2,3*], Jihong Wen[1*], Li Cheng[3], Dianlong Yu[1], Hongjia Zhang[1], Peter Gumbsch[2,4*]

[1]College of Intelligent Science and Technology, National University of Defense Technology, Changsha, Hunan 410073, China.

[2] Institute for Applied Materials, Karlsruhe Institute of Technology (KIT), 76128 Karlsruhe, Germany.

[3]Department of Mechanical Engineering, Hong Kong Polytechnic University, Hong Kong, China.

[4]Fraunhofer Institute for Mechanics of Materials IWM, 79108 Freiburg, Germany.

Correspondence to: peter.gumbsch@kit.edu (P.G.), xinfangdr@sina.com (X.F.), wenjihong@vip.sina.com (J.W.)

This file includes:

Legends for 5 Supplementary Videos

Figs. S1 to S23 partially with explanatory text

Supplementary Table 1

Supplementary Table 2

In this file, the supplementary notes and figures are not divided into different sections. We explain the figures with supplementary notes where the figures appear.

Legends for Supplementary Videos

Supplementary Video 1 | Introduction of the metamaterial based on Taiji gears. This movie shows the robust tunability and high stability of the proposed metamaterial under a large force.

Supplementary Video 2 | Actuation of the micro metamaterial consisting of Taiji gears.

Supplementary Video 3 | Integrated 3D printing process of the macro metamaterial consisting of a planetary gear system.

Supplementary Video 4 | Actuation of the macro metamaterial consisting of planetary gears.

Supplementary Video 5 | Shape morphing metamaterial. This movie shows the structure, manufacturing process and the protected shape morphing of this metamaterial.

Supplementary Figures and Explanatory Notes

Briefing on involute gears: All gears adopted in this paper are *planar involute gears* widely used in engineering. In an involute gear, the tooth profile follows an involute curve. Parameters for involute gears are stated in multiple standards e.g., ISO 1328. The size of a gearwheel is defined by the *reference pitch diameter*, D. The *diameter* stated in the main text refers to the pitch diameter. Three important parameters which determine a gear configuration are *pressure angle* α, *module* m and *number of teeth* z. $D=mz$. The *module* is a measure of the tooth size. The *tooth thickness* t_{to} used in this paper is defined as the arc length of the solid tooth on the pitch circle, thus $t_{to}=\pi m/2$. For standard gears, the tooth height is $h_t=2.25m$. The contact connection between each pair of gears is referred to as *engagement*, or *meshing*. To ensure a perfect match among the teeth of gears, m and α must be equal. In this paper, a standard value of $\alpha=20°$ is chosen for all gears.

Node constraints: A connection node has two constraints: $\Delta\varphi$ and Δs. $\Delta\varphi$ (Δs) denotes the relative rotation angle between the two objects (relative displacement between two points) connected by the node. For fixed nodes, $\Delta\varphi=0$ and $\Delta s=0$. For hinged nodes, $\Delta\varphi$ can vary in a specified range (i.e., partly free) and $\Delta s=0$. For a gear meshing node, $\Delta\varphi$ is completely free and $\Delta s=D(1\text{-}\cos\theta)$, where D denotes the pitch circle of the gear and θ denotes the rotation angle in the out-of-plane direction (see Fig. 2 in the main text).

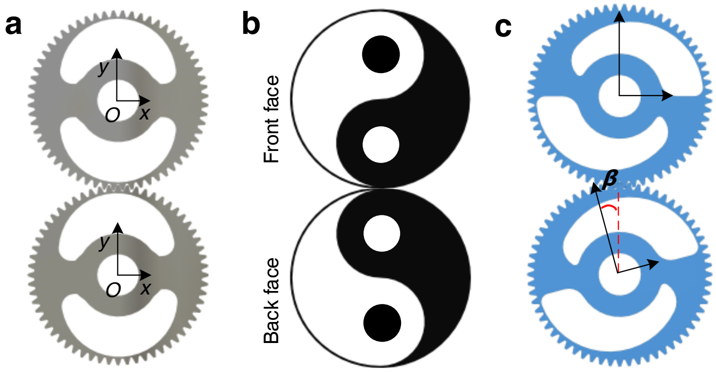

Supplementary Fig. 1 | Inspiration pictures. (**a**) A pair of meshing gears whose hollow section is symmetric about planes xoz and yoz. The origin point O lies at the center of each circle. Z-axis is the out-of-plane direction. (**b**) Front and back faces of the Taiji pattern. The Taiji pattern is centrosymmetric (or axisymmetric) instead of plane-symmetric. The spiral directions on the front and back faces are opposite: the polarity is generated. (**c**) The meshing between two Taiji gears for negative polarity. Angle β is the difference between the two local coordinates.

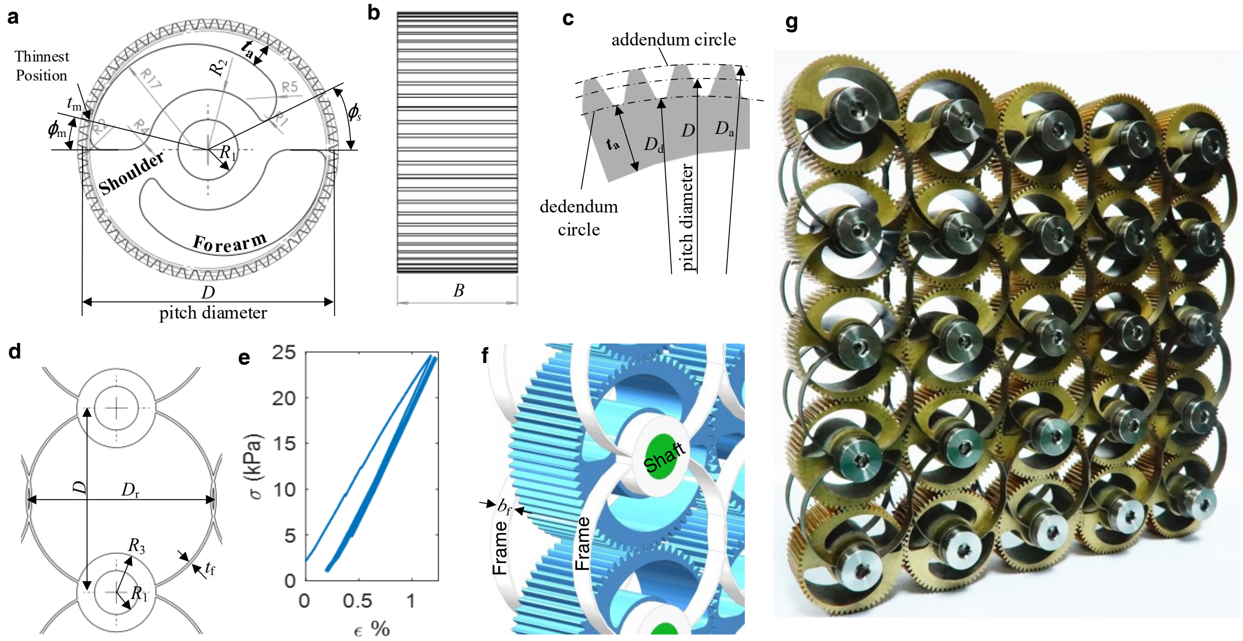

Supplementary Fig. 2| Model design. (**a-c**) Parameters of a Taiji gear. The radius of the central hole is R_1=5 mm. R_2=10 mm is the radius of the inner circle of the irregular-shaped holes. (**d**) Frame structure. (**e**) Experimentally measured strain-stress curves of the frame. (**f**) Assembly of gears and frames with shafts. (**g**) Photo of the test sample consisting of 5×5 Taiji gears in Fig. 2.

Supplementary Figs. 1-3 show the metamaterial based on Taiji gears. The definition of the *arm stiffness* k_{arm} and that of the *contact stiffness* of the meshing teeth k_{tooth} are illustrated in Supplementary Fig. 3. As shown in Supplementary Fig. 2, for the Taiji gear, m=0.7 mm and z=60. ϕ_0=360/z=6°. The angle difference β between two local coordinates (Supplementary Fig. 1c) is $\beta=n\phi_0/2$, where the integer $n\neq0$ and ϕ_0=360°/z, denoting the central angle of each tooth. The width of the gear is B=20 mm. The Taiji pattern is axisymmetric (Supplementary Fig. 1). The solid part of the Taiji gear forms two elastic arms whose radial thickness gradually decreases from the shoulder to the thinner part on the forearm. The profiles of the two irregular-shaped holes are smoothened with transition arcs. The *radial thickness* of the elastic arm t_a is determined by both D_d and the position of measurement on the forearm. t_a at the meshing point changes smoothly with θ. The two solid shoulders are the sections within an azimuthal angle ϕ_s=25°. At ϕ_m=12°, t_a reaches the minimum of 0.424 mm.

For a pair of meshing Taiji gears with opposite spiral directions, the polarity is positive since $k_{arm1}(\theta)=k_{arm2}(\theta+\beta)$. A negative polarity with $k_{arm1}(\theta)= k_{arm2}(-\theta-\beta)$ is shown in Fig. 2g. Both polarity and β affect the tunable range and the correlation between the tunable properties and θ. Positive and negative polarities are labelled as $P^+(\beta)$ and $P^-(\beta)$, respectively. $k_{arm1}(\theta)\approx k_{arm2}(\theta)$ for $P^+(3°)$.

All Taiji gears are made of brass (copper alloy) with continuum elastic modulus E_s=100 GPa, Poisson's ratio μ= 0.34 and density ρ_s=8900 kg/m^3. The frame is shaped into a two-dimensional (2D) periodic lattice structure (Supplementary Fig. 2d). The unit cell of the frame is an annulus with a diameter D_r=42.3 mm and a thickness t_f = 0.5 mm. The width of the entire frame is b_f=5 mm. Gears are periodically assembled with two frames connected by shafts. The same inner gear compactly meshes with its four neighbors. Theoretically, no clearance should exist between any pairs of meshing teeth. The meshing connection remains reliable until the stretching deformation between a pair exceeds the tooth height. The steel frame with E_s=210 GPa, μ=0.3 and ρ_s=7850 kg/m^3 is manufactured with a wire-electrode cutting machine. E_{fx}=E_{fy}=2.0±0.4 MPa (experimentally measured value, Supplementary Fig. 2e).

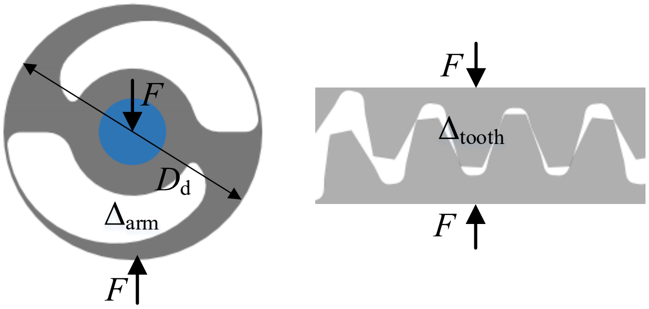

Supplementary Fig. 3 | Models to define the arm stiffness k_{arm} and the contact stiffness k_{tooth}. By applying a pair of forces F with equal amplitude but opposite direction (aligned by one radius) respectively on the shaft center and on the arm of a teeth-free gear, deformation Δ_{arm} is induced. The arm stiffness is k_{arm}=F/Δ_{arm}. For contact stiffness, only meshing teeth are considered (right panel). Applying equal and opposite forces F on the top and the bottom surfaces leads to a change in the distance between the two surfaces Δ_{tooth}. The contact stiffness is defined as k_{tooth}=F/Δ_{tooth}.

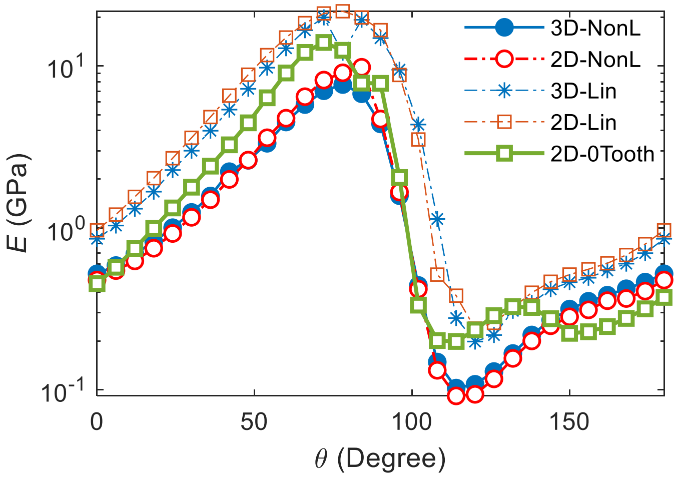

Supplementary Fig. 4 | Finite element simulation. Dependence of the Young's Modulus E on the driving rotation angle θ obtained from finite element simulations with 2D and 3D setups and different contact properties: 'Lin' stands for the bonded meshing gears while 'NonL' represents the frictionless contact; '2D-0Tooth' denotes the simplified 2D model in which all teeth are removed.

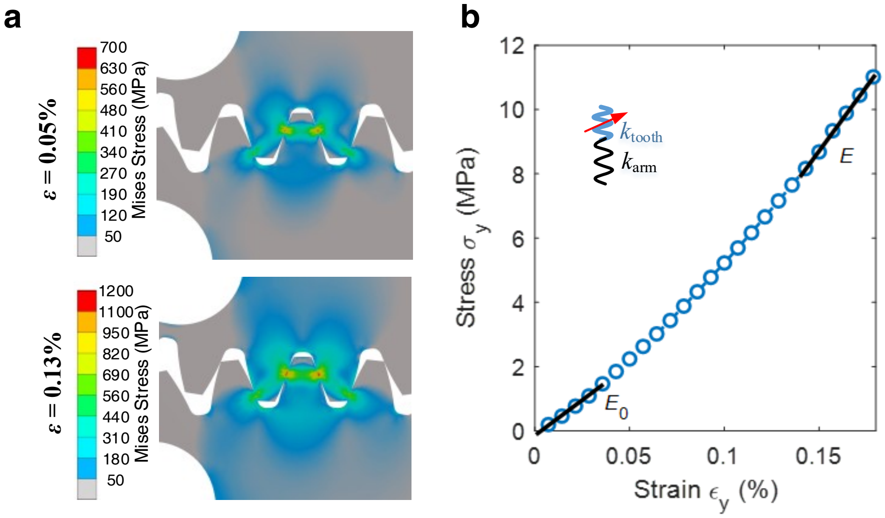

Supplementary Fig. 5 | Contact nonlinearity between meshing gears. (a) Meshing contact between two gears. The position of the contact points also depends on θ. (**b**) Typical deformation curve of a pair of gears under compressive loading. Here, E_0 and E denote the slopes of the strain-stress curve at small and relatively large strain, respectively. Nonlinearity appears due to the contact between the curved surfaces on teeth.

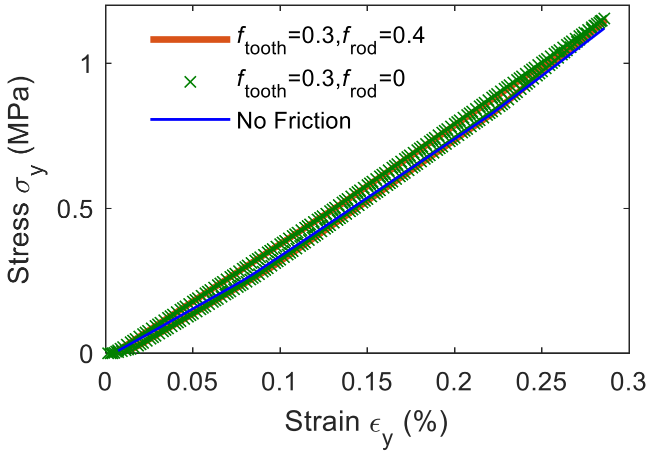

Supplementary Fig. 6 | Damping properties. Strain-stress curves in different cases. All simulations are performed with the 3D finite element model for P^+(β=3°) and θ=0. Here, f_{tooth} is the frictional factor between meshing teeth, and f_{rod} is the factor between the shaft and the cylindric hole. Results show negligible influence of f_{rod} on the hysteresis behavior.

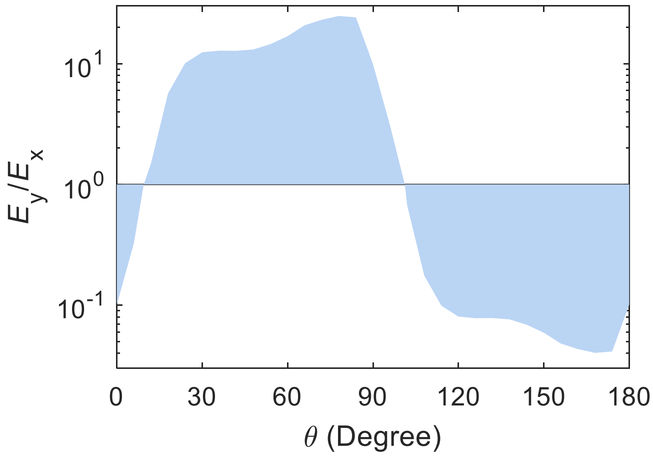

Supplementary Fig. 7 | Value of E_y/E_x. It indicates the anisotropic property in the orthogonal directions.

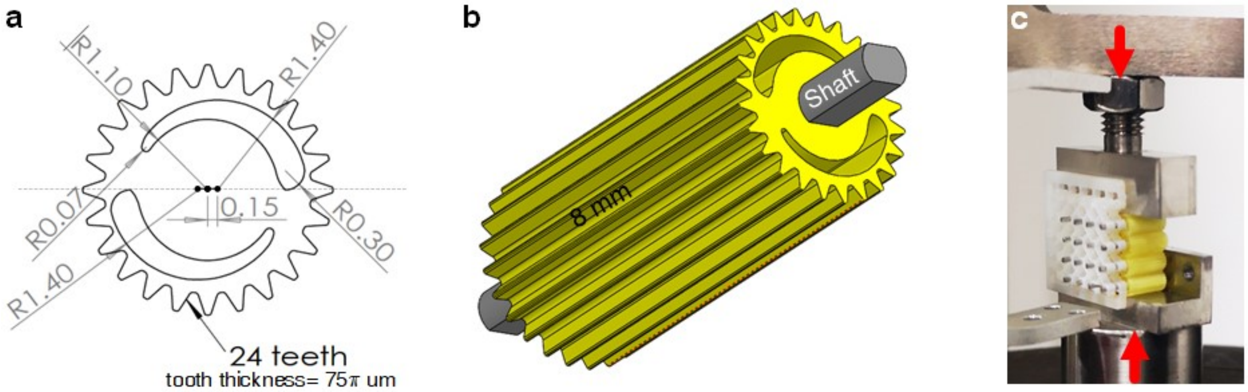

Supplementary Fig. 8 | Model for the micro metamaterial consisting of Taiji gears. (**a**) Structural parameters of the micro Taiji gears. Here dimensions are labeled in unit mm. (**b**) Picture of a gear. A hole is fabricated at the center of the gear, and a micro steel shaft is used to connect the gear to the polymer frame or the actuation motor. (**c**) Picture of the experimental setup. We take a specimen with 4×4 micro gears to measure the compressive Young's modulus.

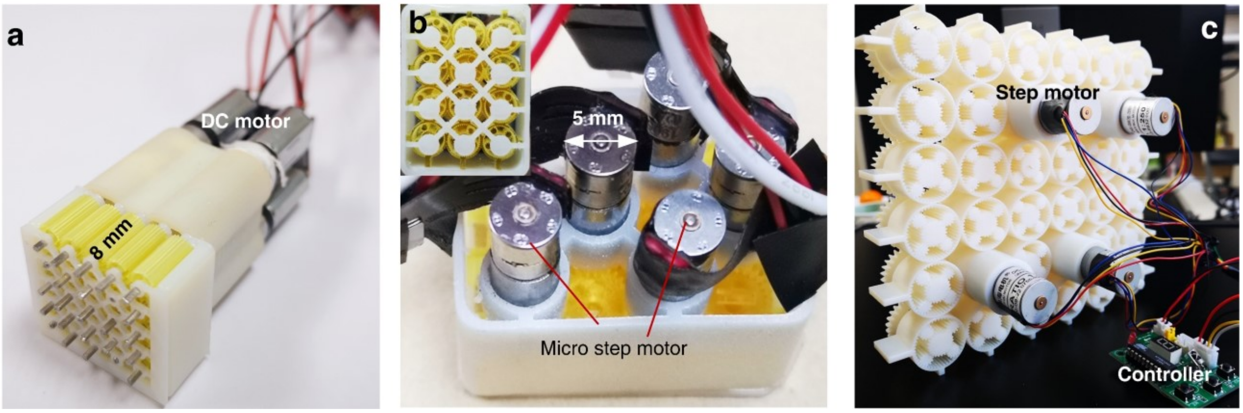

Supplementary Fig. 9 | Actuation of typical specimens with motors. **(a)** Specimen consisting of 5×5 micro Taiji gears. (**b**) Specimen consisting of 3×4 micro planetary gears. (**c**) Specimen consisting of 6×6 planetary gears. Additional discussions on this figure are provided in Methods. Watch the supplementary Videos for more details.

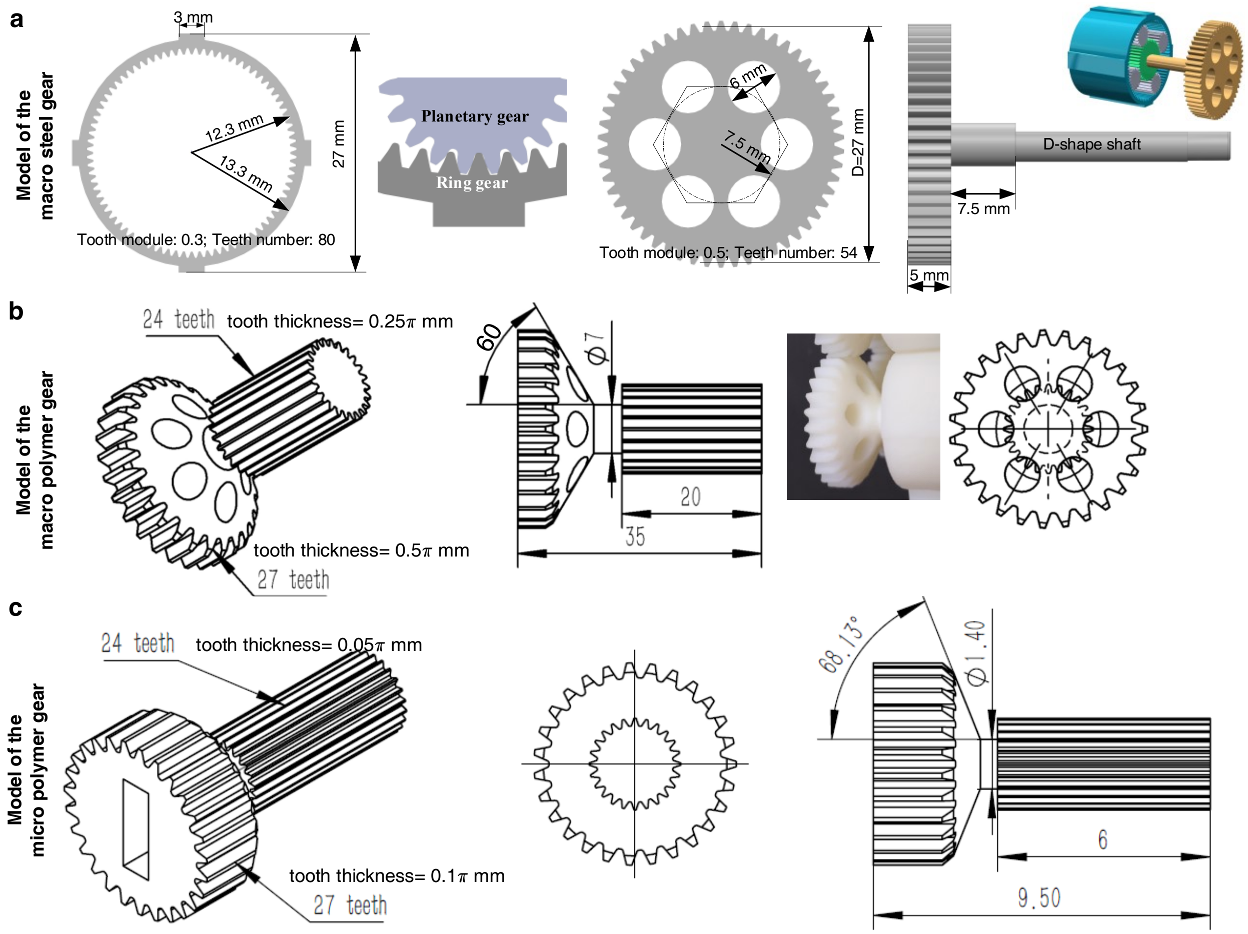

Supplementary Fig. 10 | Gear models used in the metamaterials based on planetary gear systems. (a) Model of the macro steel gears. This panel also shows the engagement between the ring and the planetary gears, alongside the connection between the transmission and sun gears. A shaft passes through the central hole on the sun gear to connect with the transmission gear. (**b**) Model of the macro polymer gear for integrated 3D printing. (**c**) Model of the micro polymer gear for integrated 3D printing. Here, a groove is fabricated on the transmission gear to connect with the actuation motor.

A planetary gear system consists of a central sun gear, an outer ring gear, and four solid planetary gears inserted between the sun and the ring. Different from other gears with outer teeth, the teeth on the inner side of the ring have concave surfaces that are conformal with the convex teeth of the outer gears (Supplementary Fig. 10a). The radii of the ring, sun and planetary gears are R_{in}, r_{sun} and r_p, respectively. The rotational transmission relationship between these gears is given by: $\theta_{ring}R_{in}+\theta_{sun}r_{sun}=\theta_{pr}(R_{in}+r_{sun})$, with the rotation angle of the ring θ_{ring} being zero here. The lattice constant of these metamaterials is $a_x=a_y$. For a given tooth module of a gear m, the tooth thickness on the pitch circle is $h_t=\pi m/2$. Corresponding parameters are listed in Supplementary Table 1. Other parameters are labeled in Supplementary Fig. 10.

Supplementary Table 1 | Parameters of the metamaterials based on planetary gear system.

Parameters	Macro steel specimen	Macro polymer specimen	Micro polymer specimen
Radius of ring gear, R_{in}	12 mm	12 mm	2.4 mm
Radius of sun gear, r_{sun}	6 mm	6 mm	1.2 mm
Radius of planetary gear, r_{p}	3 mm	3 mm	0.06 mm
Ring thickness, t_{r}	1.0 mm	1.1 mm	0.3 mm
Thickness in z-axis of sun gear, B	20 mm	20 mm	6 mm
Tooth module *m* of sun gear	0.3 mm	0.5 mm	0.1 mm
Tooth module *m* of ring gear	0.5 mm	1.0 mm	0.2 mm
Lattice constant	27 mm	27 mm	5.4 mm

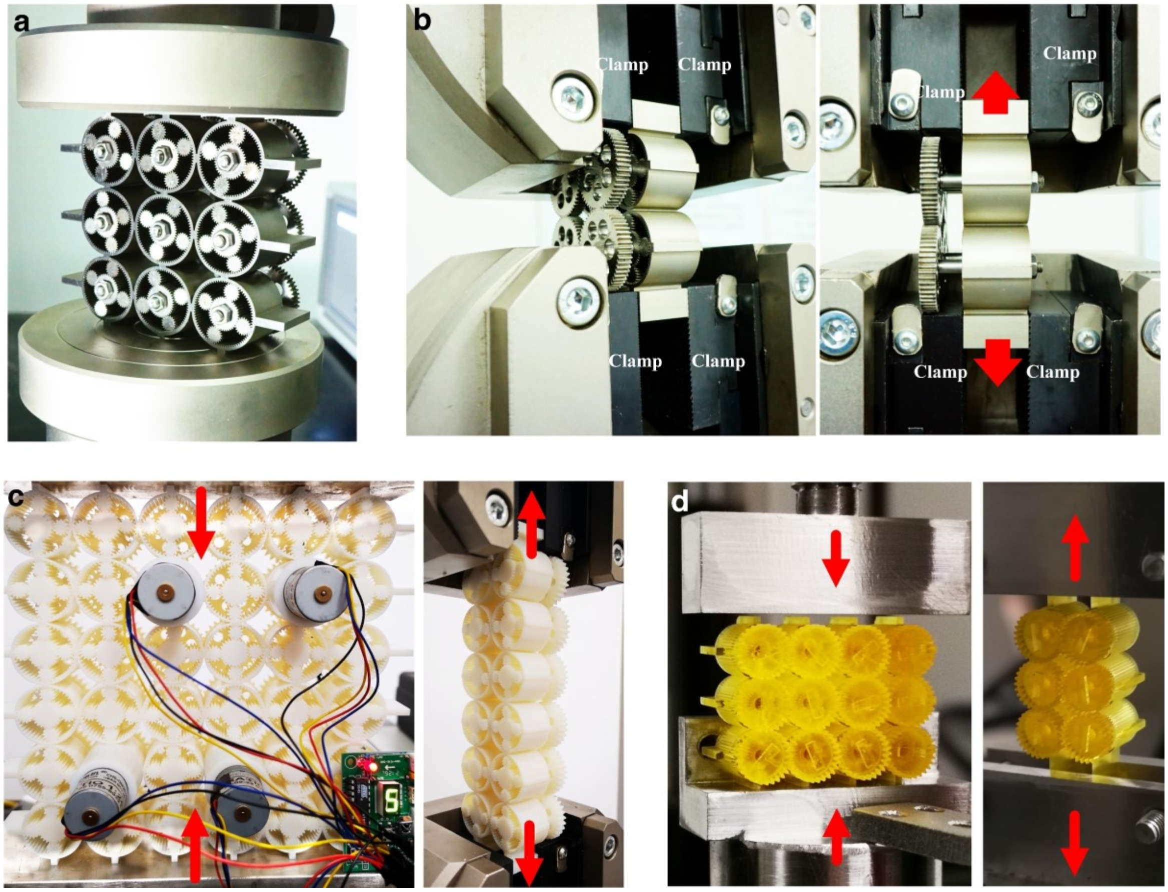

Supplementary Fig. 11 | Compression and tension experiments on the metamaterials based on planetary gear system. (**a, b**) All-steel metamaterials. (**c**) Macro polymer metamaterials. (**d**) Micro polymer metamaterials. In compression, the compressive load is applied by two parallel planes. In tension, the tails of the sample are fixed inside two pairs of clamps, through which the tensile load is applied.

For the all-steel metamaterial, a 3×3 specimen is manufactured to measure the tunable compressive Young's modulus, and a 2×2 specimen for the tunable tensile modulus. A smaller sample is used in tension because of the size limitation of the clamps. The tails extending from the blocks on the rings are used to apply tensile loads. For the macro polymer metamaterial, the 6×6 and 2×6 specimens are manufactured for measuring compressive and tensile Young's modulus, respectively. The long and narrow 2×6 specimen used in tension can eliminate the influence of boundary conditions explained in Supplementary Fig. 13. For the micro polymer metamaterial, the 3×4 and 2×3 specimens are manufactured for measuring compressive and tensile Young's modulus, respectively. The number of unit cells are limited by the 3D printer.

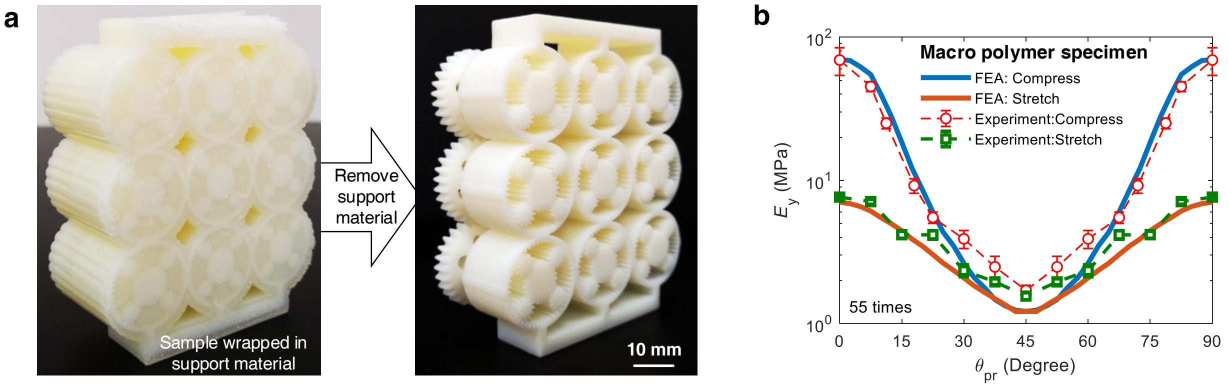

Supplementary Fig. 12 | (a) Integrated manufacturing of the macro-metamaterials consisting of planetary systems. Details for manufacturing are stated in Methods-Integrated manufacturing in the main text. (**b**) Experimental results on the 6×6 macro polymer specimen.

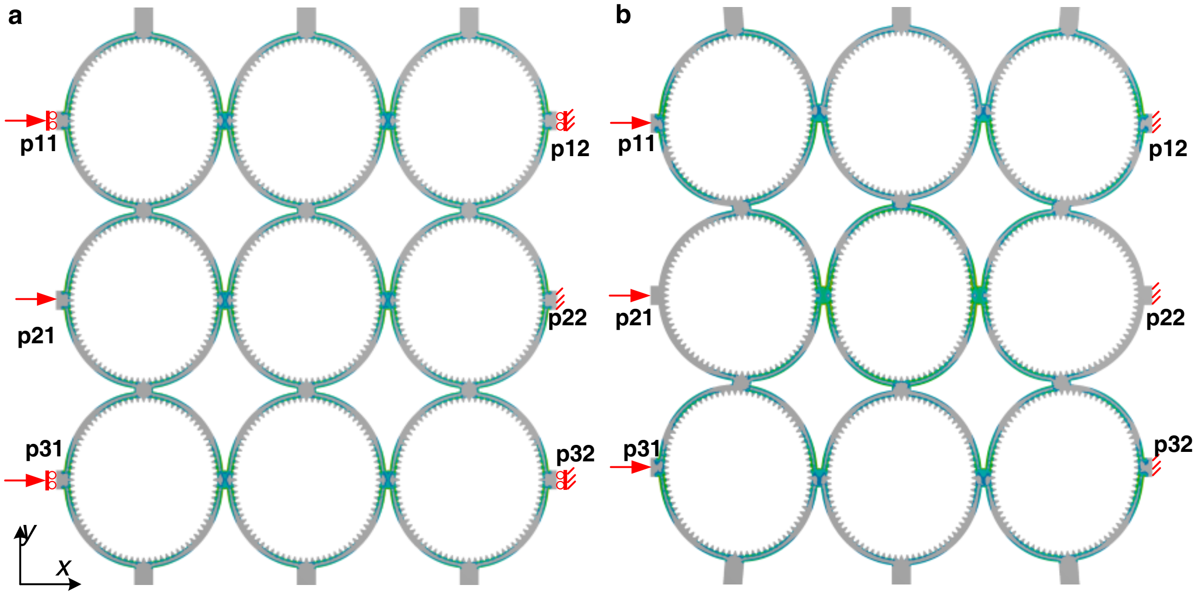

Supplementary Fig. 13 | FEA simulations of the 3×3 frame under free and fixed boundaries. This simulation corresponds to the case θ_{pr}=45° on which occasion gears inside the frame are not bearing load. Here the deformation is enlarged by 100 times for better visualization. Planes **p*ij*** are the six positions for applying load and boundary constrains that are realized with displacement fields (u, v), i=1,2,3; j=1, 2. Here we use the coordinate shown in (**a**). All subscripts *i, j* represent the displacements of corresponding planes, p*ij*. (**a**) Boundary conditions are: $u_{22}=v_{22}= v_{21}=0$; $u_{12}=u_{32}=0$, $u_{11}=u_{b1}=u_{31}$=specified deformation, but other components v_{11}, v_{31}, v_{12} and v_{32} are free. (**b**) Different from boundary conditions in (**a**), the y-axis deformation at all planes **p*ij*** is constrained to 0. Boundary conditions in (**a**) and (**b**) give the equivalent Young's modulus E_f=82 MPa and 120 MPa, respectively.

For the metacell in Fig. 3, deformation in x-axis is free when applying a force in y-axis, which gives the equivalent Young's modulus $E(\theta_{pr}$=45°)=82 MPa, equal to the FEA result in panel (**a**). Experiment yields $E(\theta_{pr}$=45°)=112 MPa, that is close to the FEA result in panel (**b**). Differences arise from the boundary conditions. As shown in Supplementary Fig. 11, when applying the compressive (tensile) loads on the metamaterial through planes (clamps) in y-axis, the x-axis deformation is affected by the frictional force between the metamaterials and the planes (or clamps). Therefore, the boundary constrains in experiments are identical with those in panel (**b**), and thus, the experimental results near θ_{pr}=45° are higher than the theoretical value with perfect boundary conditions in panel (**a**).

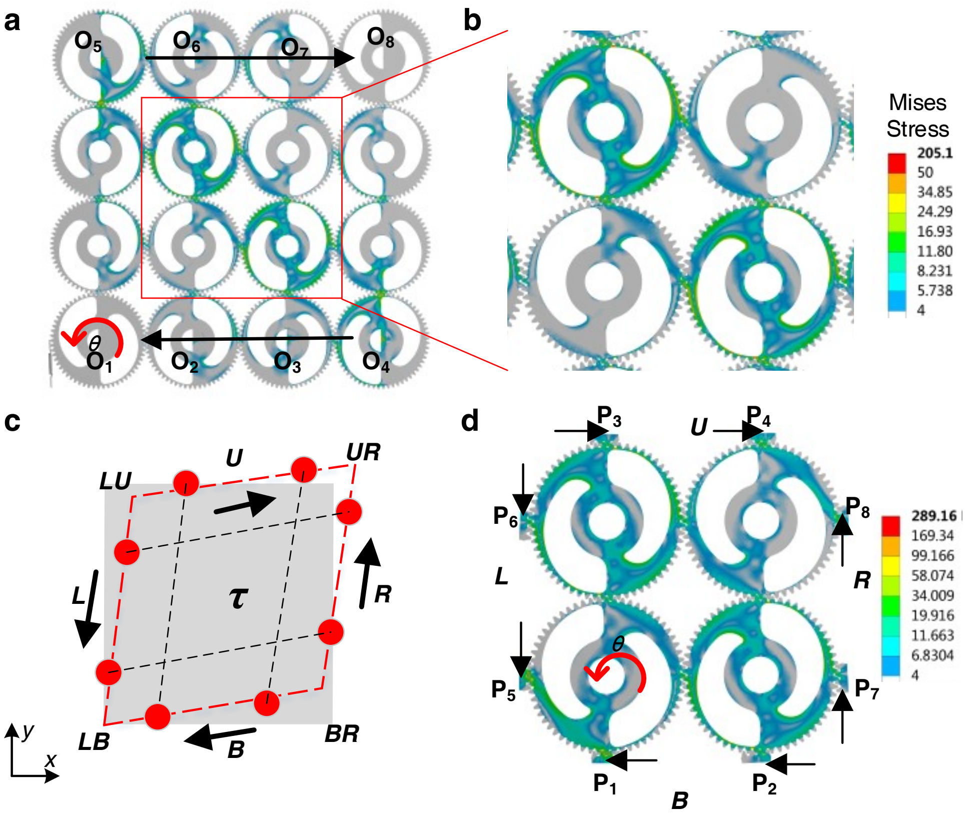

Supplementary Fig. 14 | FEA model for shear modulus of the metamaterial based on Taiji gears in interlock state. (**a**) The finite 4×4 model. (**b**) The enlarged picture from (**a**). (**c**) Sketch diagram for shearing state. (**d**) The model with periodic boundary conditions (PBCs) on a metacell. The rotation angle is θ=90° in (**a, b, d**). Arrows in (**c**, **d**) label the applied PBCs on the eight meshing positions P_1~P_8. Red points in (**c**) represent the eight positions P_1~P_8 in (**d**). 'L, R, B, U' symbolize the left, right, bottom and upper edges (or faces in 3D). LB, BR, LU, and UR are four virtual vertices of the deformed parallelogram.

In panel (**a**), we fix the lower row of gear centers (i.e., points O_1~O_4) and apply displacement fields $u\neq 0$ and v=0 to the upper row of gear centers (i.e., points O_5~O_8). The homogenized shear strain is $\gamma=u/L_y$ and $L_x=L_y=3D$=126 mm. When applying this shearing boundary condition, every gear inside the metamaterial is locked by four shear forces arising from its neighbor gears, and those forces are balanced so that gear rotation is prohibited, i.e., *shear interlock* appears. In shear interlock state, gear teeth at loading positions are bended, and the elastic arms between the two shear forces are squeezed or stretched. However, gears at boundaries (corners) only bear loads at three (two) positions, i.e., free edge effects appear. Due to the free edge effects, calculating shear modulus G by applying this boundary condition requires very large number of metacells, the generalized shear stiffness G' also depends on the size of the gear lattice.

Instead, we calculate the shear modulus from strain energy[1]. The strain energy density function denotes $W=C_{ijkl}\varepsilon_{ij}\varepsilon_{kl}/2$ with C_{ijkl} being the stiffness tensor and ε_{ij} the strain tensor. Our metamaterial based on Taiji gears is anisotropic in orthogonal directions. In the plane stress state, if $\varepsilon_{11}=\varepsilon_x=0$, $\varepsilon_{22}=\varepsilon_y=0$ but the shear strain tensor $\varepsilon_{12}=\varepsilon_{21}=\gamma/2$ is nonzero, the strain energy density becomes $W=G\gamma^2/2=2G\varepsilon_{12}^2$. This allows one to evaluate shear modulus, $G=W/2\,\varepsilon_{12}^2$. Implementing this method requires applying periodic boundary conditions (PBCs) on a metacell, as shown in panel (**d**). We put eight gear blocks at positions P_1~P_4, respectively, and boundary conditions are applied on the blocks instead of the gears here. The shear state is applied by enforcing the following PBCs on the displacement components (u_i, v_i) on the left, right, bottom and upper edges (P_1~P_8):

$$u_R - u_L = 0,\ v_R - v_L = \varepsilon_{12}a_x,\ u_U - u_B = \varepsilon_{12}a_y,\ v_U - v_B = 0\ .$$

Here the lattice constants are $a_x=a_y=2D=84$ mm. To establish these conditions in FEA using ANSYS, the displacement fields of the four virtual vertices of the parallelogram in panel (**C**) are regarded to be following:

LB (0, 0); BR (0, $\varepsilon_{12}a_x$); LU ($\varepsilon_{12}a_x$, 0); UR ($\varepsilon_{12}a_x$, $\varepsilon_{12}a_x$).

Then, the displacements at the eight blocks P_1~P_8 are determined by the parallelogram. For example, the displacement field of P_8 is ($3\varepsilon_{12}a_x/4$, $\varepsilon_{12}a_x$). In simulations, we specify the average strain $\varepsilon_{12}=0.04/84$. With these PBCs, the distribution of the stress in panel (**d**) is symmetric, and this stress contour is similar with that in the inner gears of finite structure in panel (**b**). The difference in stress value, especially in the top-right gear and the left bottom gear in (**b**, **d**), arises from the different boundary conditions, especially the free edge effect in panel (**a**).

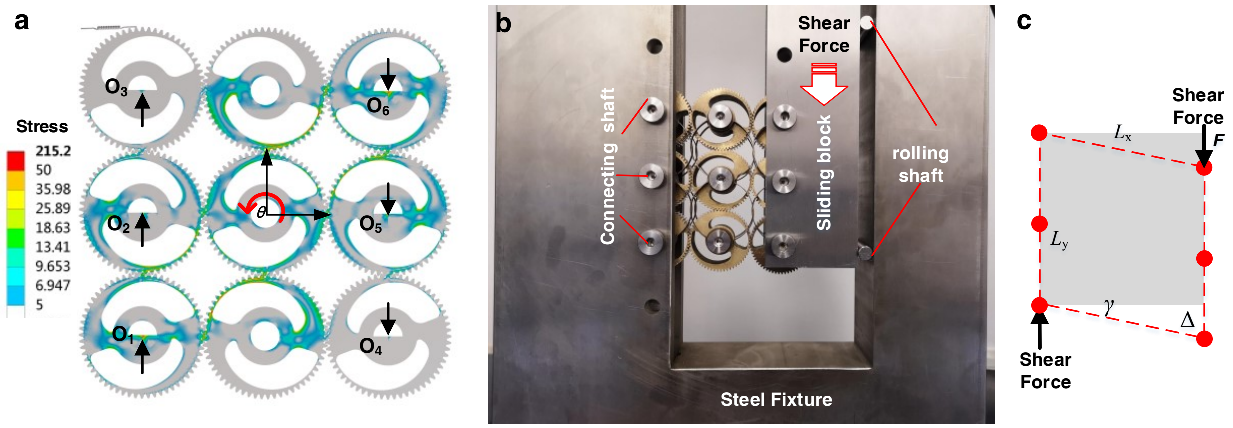

Supplementary Fig. 15 | Simulation and experiment about the shear stiffness in interlock state. A prototype consisting of 3×3 Taiji gears is used. (**a**) Stress contour in FEA simulation. (**b**) Photo of the experimental set-up for shear test with samples installed in the fixture. (**c**) Mechanical model for measuring the shear stiffness using the set-up in (**b**).

In panel (**a**), we fix one row of gear centers (i.e., points O_1~O_3) and apply displacement field $v\neq0$ and $u=0$ to the other row of gear centers (i.e., points O_4~O_6). The free edge effect appears again in the outer eight gears in this model. In (**b**), the sample is connected to the steel fixture on the left and to a sliding block on the right, both through a column of shafts. When applying a shear force F_y onto the top of the sliding block, it slides along a fixed guiding rail with two rollers, causing deformation in y axis for shear loading. The influence of friction is greatly reduced by the rollers, and the influence of the lateral deformation in x-axis is reduced by the connecting shafts. Therefore, this experimental set-up is designed to obtain the shear state: $\varepsilon_x=0$, $\varepsilon_y=0$, and the shear stain $\gamma=\Delta/L_x$, where the measured shear deformation Δ is the relative slippage between loading and fixing positions. The shear stiffness is $K_{shear}=F_y/\Delta$. The equation for the *generalized shear stiffness* $G'=K_{shear}/B$ which is identical to the formula for shear modulus $G=\tau/\gamma$ because $L_x=L_y$, and the shear stress $\tau=F_y/BL_y$.

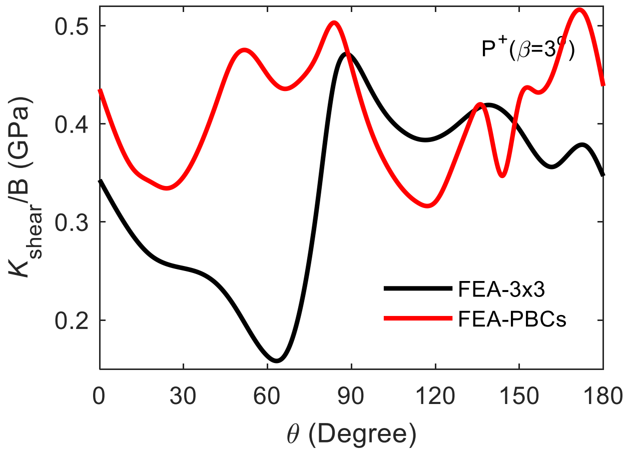

Supplementary Fig. 16 | Tunable shear stiffness obtained with different FEA models when changing the rotation angle *θ*. 'FEA-PBCs' represents the model with periodic boundary conditions shown in Supplementary Fig. 14e. 'FEA-3×3' represents the 3×3 model shown in Supplementary Fig. 15a. As analyzed in Supplementary Fig. 14a and Supplementary Fig. 15a, the value difference arises from the free edge effects in the finite structures.

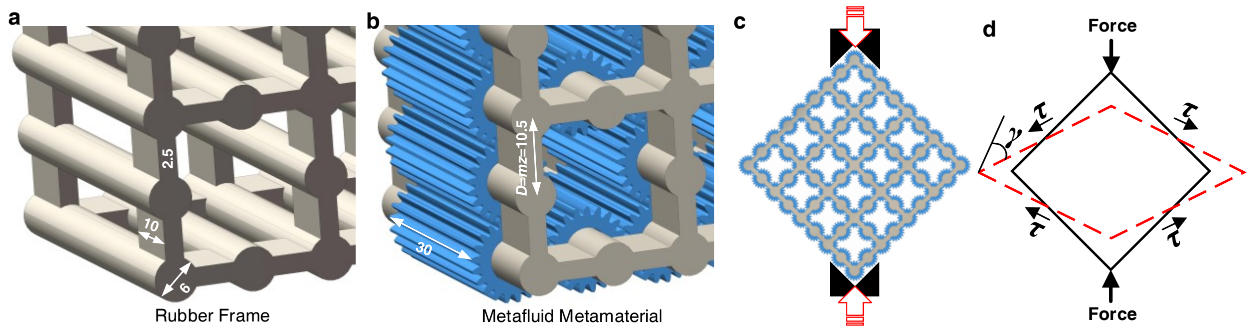

Supplementary Fig. 17 | Structure and testing of the shape-morphing metamaterial. (**a**) Rubber frame consisting of shafts and beams. The thickness and the width of the rubber beam are 2.5 mm and 10 mm, respectively; the diameter of the shaft is 6 mm. (**b**) Local structure of the metamaterial made of gears (blue) and rubber frame (grey). The width of the aluminum alloy gear is 30 mm. Rubber shafts pass through the gears' central hole for assembly. (**c, d**) Schematic diagram and experimental set-up used for shear modulus testing under diagonal compression. The sample is put in two right-angle grooves, and the load from the testing machine is directly transferred to the sample.

This metamaterial is composed of aluminum alloy gears and three-dimensional (3D)-printed rubber frames. As shown in Supplementary Fig. 18d, for perfect meshing between 3 identical gears, the number of tooth z must meet the condition $(2n+1+0.5)\times360°/z=60°$, i.e., $(2n+1+0.5)\times6=z$, here n and z are integers. In our design, z=21 meets this condition. Other parameters for the gear are: tooth thickness h_t=0.25π mm, D=10.5 mm, B=30 mm. The elastic modulus and Poisson's ratio of the rubber are 30 MPa and 0.4, respectively.

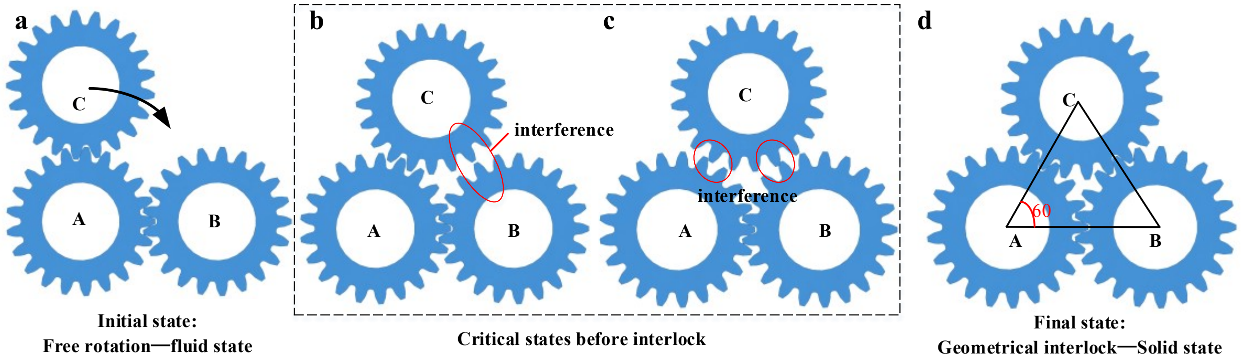

Supplementary Fig. 18 | Meshing process of three gears in the prototype from the ultrasoft state to solid state. (**a**) Initial ultrasoft state. (**b**, **c**) Two critical state before interlock. (**d**) Final interlock state behaves as solid.

The shear modulus shows oscillations in Fig. 4c in the main text. The phenomenon arises from the critical states before final interlock. The meshing process is shown in Supplementary Fig. 18. Though the three gears can perfectly mesh and form the geometrical interlock, there are interferences between their teeth just right before the interlock (see panel b, c). Therefore, from the critical states in (b, c) to the interlock state in (d), the teeth in contact are bent and the relative positions between three gears is slightly adjusted, so that gear C can "slide" into the teeth of gears A and B. Such "sliding" leads to the oscillation in Fig. 4f. Every three neighbor gears inside the metamaterial can generate this critical state. Moreover, because the deformation of the rubber frame is not uniform in practice, not all "sliding" occur simultaneously. The "successively sliding" instead of the "simultaneously sliding" leads to several oscillations. At last, the geometric interlock between every three gears occurs, and the metamaterial behaves as a solid. Reducing the number or height of tooth can alleviate or eliminate these oscillations.

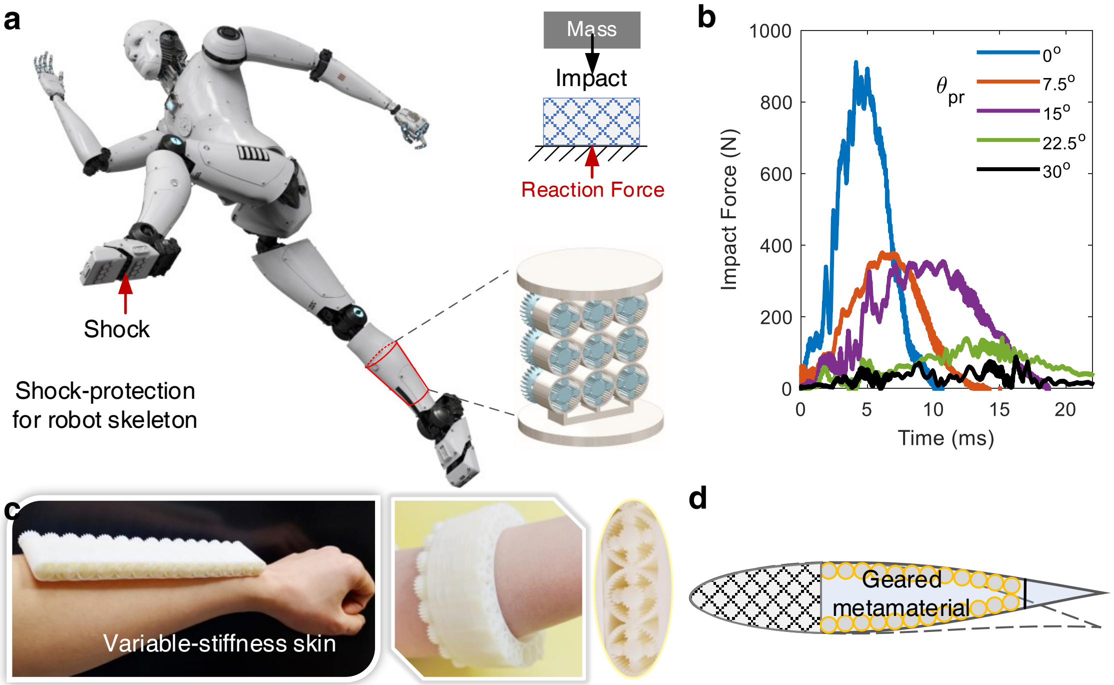

Supplementary Fig. 19 | Potential applications of gear-based metamaterials. (**a**) Schematic diagram of a shock isolator in a robotic skeleton. The isolator can be embedded into human legs. This picture comes from Ref. 5 here. (**b**) Measured impact force transferred from an impact mass to the bottom of the isolator under different rotation angles θ_{pr} of the planetary gear-based metamaterial. The setup used for the impact test is shown in Supplementary Fig. 20. (**c**) Variable-stiffness skins consisting of planetary gear-based metamaterial. (**d**) Schematic diagram of an adaptive shape morphing wing. The shock isolator and variable-stiffness skin are all printed with a polymer that can sustain large deformation (its Young's modulus is about 2.0 GPa).

As shown in shown in Supplementary Fig. 20, we perform impact tests on an isolator/actuator consisting of 3×3 polymer planetary gears. As illustrated in Supplementary Fig. 19b, the impact force transferred from the impact mass to the bottom of the isolator can be reduced by 12 times by tuning the rotation angle θ_{pr} from 0 to 30°. The results show that a tunable-stiffness leg/actuator in robot can offer high stiffness to stably support heavy load while walking, and can tune to low stiffness for shock-protection while jumping/running.

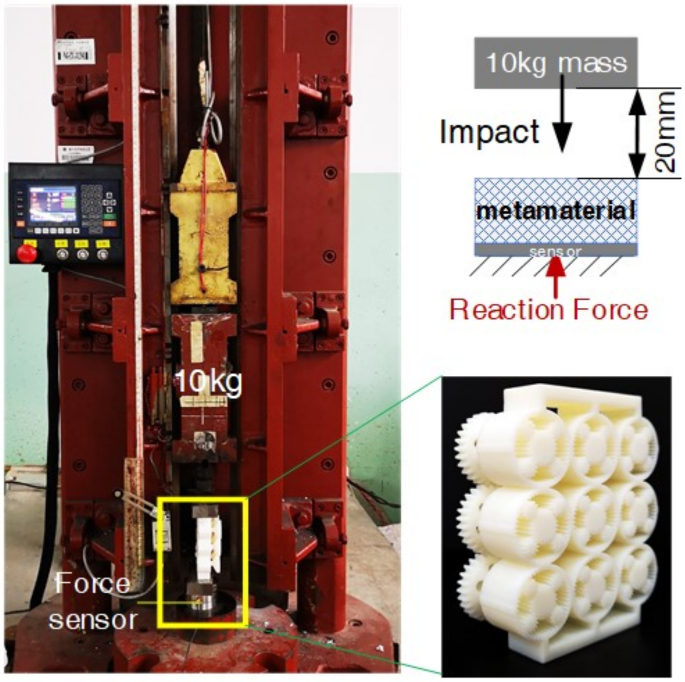

Supplementary Fig. 20 | Experimental setup for impact test: hammer dropping experiment. A 10 kg steel mass is lifted by a string. The separation of this mass is controlled by an electromagnet. A metamaterial consisting of 3×3 polymer planetary gears is used as a shock isolator. The configuration of this metamaterial is identical with the one shown in Fig. 3d. The isolator is put on a steel block. A force sensor is set between this block and a rigid plane to measure the contact force during the collision. The distance between the mass and the polymer isolator is set to be 20 mm.

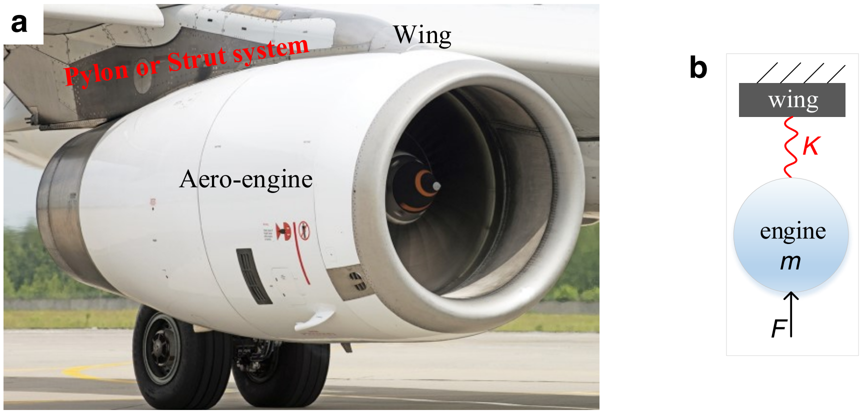

Supplementary Fig. 21 | Potential application on the aeroengine pylon system. (**a**) Aero-engine and aircraft. (**b**) Simplified model for the aero-engine system. Here, the mass of engine m connects to the fixed wing/fuselage through a spring with stiffness K.

Tunable-stiffness aero-engine pylon system: A pylon system is usually used to mount an aero-engine on the wing/fuselage of an aircraft. While transmitting engine thrust, it also transmits vibration caused by asymmetrical loading of the rotor disc and blade tip shocks, to/throughout the airframe. The induced vibration yields cabin noise and airframe fatigue, even compromises the aircraft safety. Vibration isolation is therefore a critical function for engine suspension. Pylon stiffness K has paramount impact on the dynamic interaction between the engine and the aircraft wing/fuselage [2, 3].

The engine-pylon assembly can be simplified as a one-degree-of freedom system shown in Supplementary Fig. 21b. The static deformation of the pylon system depends on its static stiffness K. The vibration transmitted from the engine to the wing also depends on the stiffness K that is tunable by rotation gears. Increasing the stiffness can reduce the static deformation for higher safety. However, decreasing the stiffness can reduce the vibration transmission above a critical frequency.

Current pylon design produces constant stiffness, and its stiffness is a compromise between static deformation and vibration transmission. The trend for long-lifetime and low-noise aircraft makes such compromises less acceptable. A tunable stiffness can potentially maintain the best performance and efficiency in different flight stages, which unfortunately cannot be achieved by classical materials. Our stable, significantly and robustly tunable metallic gear metamaterials can offer an ideal solution to the problem: fabricating those connectors with gear metamaterials in Figs. 2 or 3 in the main text. For example, in cruise stage where the thrust becomes smaller after taking off, the connection stiffness can be reduced to create a quieter cabin environment and longer lifetime of aircraft, only by robustly rotating gears. Reducing vibration is a major concern for aircraft design due to the demands of stability, safety and low noise[4].

Supplementary Table 2 | Potential applications

**Tunability**	**Properties**	**Potential applications**
Continuously tunable Young's Modulus by two orders of magnitude	1. Smooth tuning of stiffness & deformation by two orders of magnitude.<br>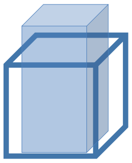<br>2. Resonant frequency changes.<br>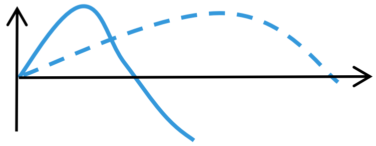<br>3. Adjustable wave transmission.<br>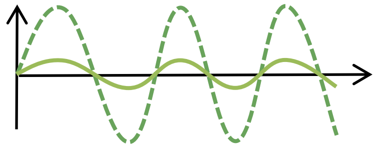	1. Real-time optimization for maximum efficiency and best performance of intelligent devices, machines or systems under complex external environment.<br>2. Variable-stiffness actuators, dampers and isolators.<br>3. Variable-stiffness muscles and gripper in robots.<br>4. Active elastic metamaterials/structures for controlling wave, vibration and noise.<br>5. Promising solution to cope with the inherent competing requirements in terms of high stiffness and reversible deformation posed by morphing in morphing aircraft or robots.
Shape morphing	1. Switching between fluid and solid states;<br>2. Diverse deformation modes (depends on boundary conditions);<br>3. Zero shear modulus	1. Morphing wings/structures that allow flexible shape changes and locking for high load-bearing.<br>2. Reprogrammable shape morphing through boundary control.<br>3. Mechanical or acoustic cloaking for object shielding, immune from detection.

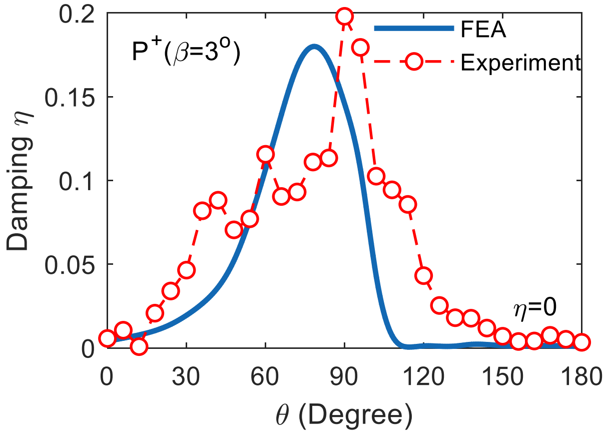

Supplementary Fig. 22 | Tunable frictional damping in the $P^+(3°)$ metamaterial based on Taiji gears. The FEA result is obtained with method mentioned in Supplementary Fig. 6. The frictional damping η reveals the energy dissipation proportion in a loading-unloading process. $\eta=S_{\mathrm{hyster}}/S_{\mathrm{tri}}$, where S_{hyster} is the area of the envelop region inside the hysteresis curves, S_{tri} is the area of the curved triangle enveloped by the loading curve and the strain axis. Under specified strain ε, the sliding friction between meshing teeth is positively related with the average equivalent stress $\sigma=E\varepsilon$. As the Young's modulus E is tunable, the damping η induced by sliding friction can also be tunable.

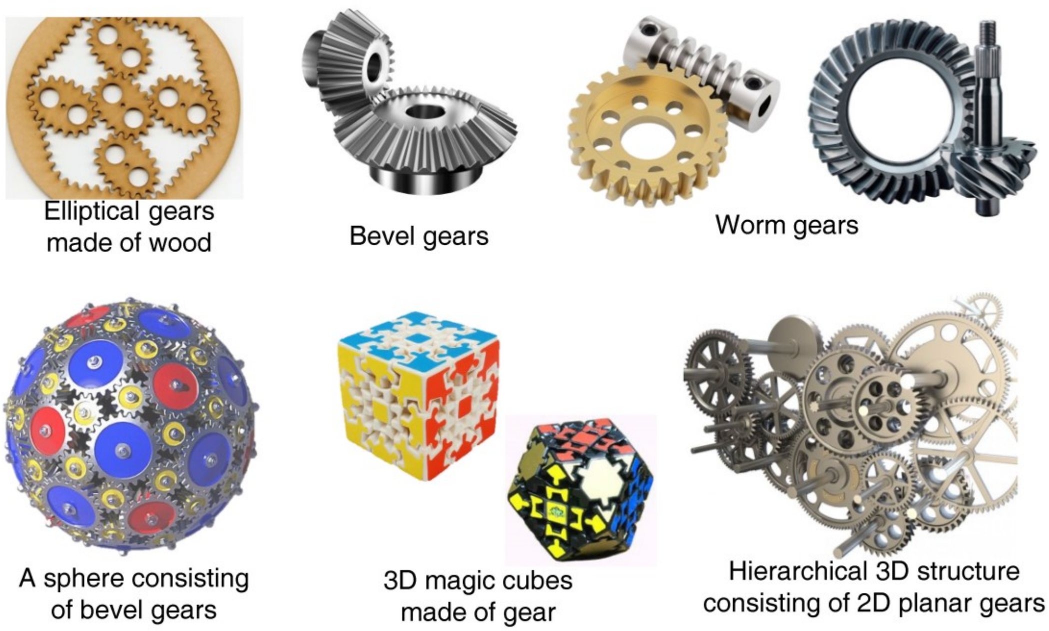

Supplementary Fig. 23 | Different types of gears and their assemblies.

References

1. Barbero, E. J. *Finite Element Analysis of Composite Materials Using Abaqus*. (CRC Press, 2013).
2. Schulze, M., Neumann, J. & Klimmek, T. Parametric Modeling of a Long-Range Aircraft under Consideration of Engine-Wing Integration. *Aerospace*. **8**, 2 (2021).
3. Coniglio, S., Gogu, C., Amargier, R. & Morlier, J. Engine Pylon Topology Optimization Framework Based on Performance and Stress Criteria. *Aiaa J.* **57**, 5514-5526 (2019).
4. Sadeghian, M. & Bandpy, M. G. Technologies for Aircraft Noise Reduction: A Review. *J Aeronaut Aerospace Eng*. **9**, 218 (2020).
5. https://www.123rf.com.cn/photo_1083814141.html
